# Controlled node growth on the surface of polymersomes†

**DOI:** 10.1039/d3sc05915d

**Published:** 2024-02-16

**Authors:** Marjolaine Thomas, Spyridon Varlas, Thomas R. Wilks, Stephen D. P. Fielden, Rachel K. O'Reilly

**Affiliations:** a School of Chemistry, University of Birmingham Edgbaston Birmingham B15 2TT UK s.fielden@bham.ac.uk r.oreilly@bham.ac.uk

## Abstract

Incorporating nucleobases into synthetic polymers has proven to be a versatile method for controlling self-assembly. The formation of strong directional hydrogen bonds between complementary nucleobases provides a driving force that permits access to complex particle morphologies. Here, nucleobase pairing was used to direct the formation and lengthening of nodes on the outer surface of vesicles formed from polymers (polymersomes) functionalised with adenine in their membrane-forming domains. Insertion of a self-assembling short diblock copolymer containing thymine into the polymersome membranes caused an increase in steric crowding at the hydrophilic/hydrophobic interface, which was relieved by initial node formation and subsequent growth. Nano-objects were imaged by (cryo-)TEM, which permitted quantification of node coverage and length. The ability to control node growth on the surface of polymersomes provides a new platform to develop higher-order nanomaterials with tailorable properties.

## Abstract 2

Nucleobase pairing was used to direct the formation and lengthening of nodes on the outer surface of polymersomes.

## Graphical abstract

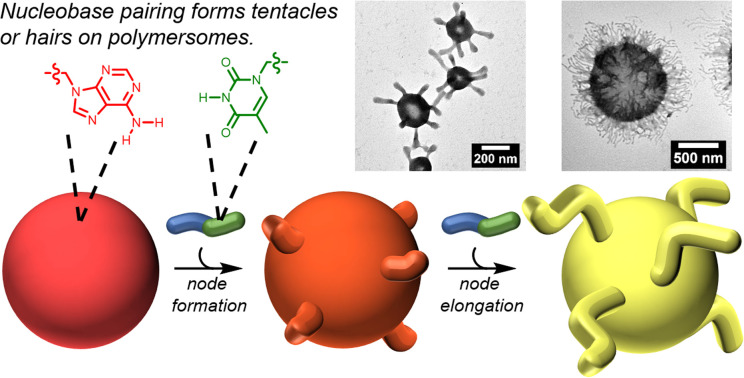

## Introduction

Biological membranes, formed from the assembly of phospholipids, adopt different shapes depending on their location within the cell.^1^ For example, membranes within the Golgi apparatus form flattened structures termed cisternae. The morphology (shape) of cisternae is optimised to maximise surface area, which in turn permits efficient spatial and temporal organisation of processes that occur on the membrane surface, such as protein glycosylation and transport.^2^

**Fig. 1:**
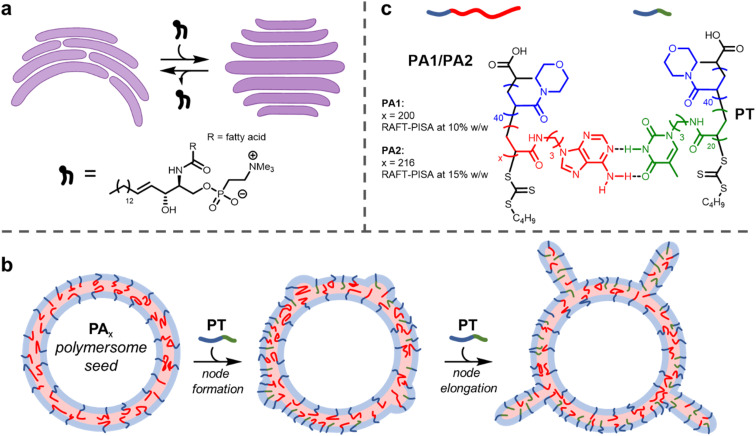
(a) Cartoon depiction of the flattening of cisternae (purple) by the insertion of SMLs. Representative chemical structure of an SML illustrating amide and alcohol groups that participate in hydrogen bonding. (b) Node growth and elongation upon addition of PT to PA1/2 polymersomes. (c) Chemical structures and complementary hydrogen bonding between polymers PA1/2 and PT.

The persistence of the anisotropic, flattened structure of cisternae partially relies on the presence of sphingomyelin lipids (SMLs) within the membranes (Fig. 1a).^3^ SMLs contain amide and alcohol functional groups, which form intermolecular hydrogen bonds with surrounding membrane components. This causes SMLs to have a strong affinity for cholesterol; when combined they associate and produce liquid-ordered domains within membranes, which provides a driving force for the flattening of cisternae.^4^ The importance of lipid composition for controlling membrane function is evident when SMLs become depleted (as occurs in Alzheimer's disease), as this causes cisternae to become more curved.^2,5^

The use of block copolymers provides the opportunity to reproduce such complex membrane behaviour in synthetic settings. The assembly of block copolymers in solution to form polymersomes, hollow vesicles delineated by a membrane formed of block copolymers, has been studied for several decades.^6–9^ Recently, a few investigations have been carried out into the deformation of isotropic (spherical) polymersomes to produce anisotropic structures.^10,11^ Global deformation of polymersome membranes (*i.e.*, changing the shape of the entire particle by adjusting membrane curvature) has been achieved by several methods, including the use of osmotic shock,^12–15^ unimer crosslinking,^16,17^ particle fusion^18–20^ or insertion of a second helical polymer.^21^ The shape of a nanoparticle often determines its properties, including therapeutic performance.^22^

It is highly desirable to engineer local changes in the shape of a nanoparticle's surface. This is because these modifications can improve biocompatibility and cellular uptake.^23^ Here, we describe a method of forming local deformations on the outer surface of nucleobase-containing polymersome membranes. We show that tentacle-like nodes can form on the surface of polymersomes upon insertion of a diblock copolymer containing a complementary nucleobase (Fig. 1b). As with cisternae, membrane deformation and consequent node formation is reliant on complementary hydrogen bonding between different membrane components.

Incorporating the programmability of nucleobase pairing into self-assembling synthetic polymers^24–28^ has been previously exploited to control nanoparticle morphology,^29–35^ bottlebrush assembly^36^ and particle surface chemistry,^37^ as well as for templated polymerisation,^38,39^ cargo delivery^40–42^ and enhancing water solubility.^43^

Notably, it has also been reported that worm-like micelles of controlled length could be synthesised from isotropic seeds (spherical micelles) using nucleobase pairing.^44^ The micelle seeds were formed by the assembly of amphiphilic diblock copolymers possessing a long thymine-containing hydrophobic block. Addition of a second polymer containing complementary adenine to the seeds produced the morphological change. First, dumbbell-type structures were formed, which further elongated to give worms of increasing length as more complementary polymer was added. Tailoring the relative length (determined by the degree of polymerisation, DP) of the constituent blocks for each copolymer was vital for the formation of anisotropic particles. When the hydrophobic adenine-containing block was shorter than the thymine-containing block (but the hydrophilic block length of the two polymers was the same), an increase in steric crowding at the hydrophilic/hydrophobic interface occurred. This crowding, brought about by a mismatch in hydrophobic chain lengths, was relieved by an increase of the surface area of this interface, resulting in particle growth in a single dimension. This growth occurred faster than isotropic swelling because it avoids the unfavourable stretching of core chains.

Here, we show this mechanism can be leveraged to induce the formation of nodes on the outer surface of polymersome seeds (Fig. 1b). When relatively small polymersome seeds were used, only a few nodes grew on each particle on insertion of the complementary nucleobase-containing polymer to form tentacle-like structures. A significantly higher number of nodes per particle formed with larger polymersome seeds to produce ‘hairy’ higher-order nanoparticles. The length of the nodes could be controlled by the amount of inserted polymer. Grafting protrusions to nanoparticles has found widespread use as a method to tailor particle properties, but is synthetically challenging.^45,46^ The facile method presented herein uses bio-inspired building blocks to direct node growth with precision.

## Results and discussion

### Synthesis of PA1, PA2 and PT

Firstly, polymersomes containing adenine functionality within their hydrophobic membrane were synthesised by aqueous reversible addition-fragmentation chain-transfer polymerisation-induced self-assembly (RAFT-PISA). As in previous work,^44^ the adenine-thymine pair was chosen in preference to guanine-cytosine. This was for two reasons: (1) adenine and thymine bind more weakly, so this pair has proved to be more useful for adaptive polymer-based systems^26,47^ and (2) the use of guanine, which has the highest self-dimerisation binding strength of the nucleobases, is avoided. Minimising the extent of self-dimerisation within the polymersomes was desirable to maximise the effect of adding a complementary polymer.

RAFT-PISA was performed using two formulations to give small PA1 and large PA2 polymersomes (Fig. 1c; see ESI† for further details). Characterisation of the constituent diblock copolymers was performed by proton nuclear magnetic resonance (^1^H NMR) spectroscopy and size exclusion chromatography (SEC) (ESI, Fig. S2 and S3†). In addition, the glass transition temperature (*T*_g_) of dried PA1 polymer was measured to be 77 °C by differential scanning calorimetry (DSC) (ESI, Fig. S6†).

**Fig. 2:**
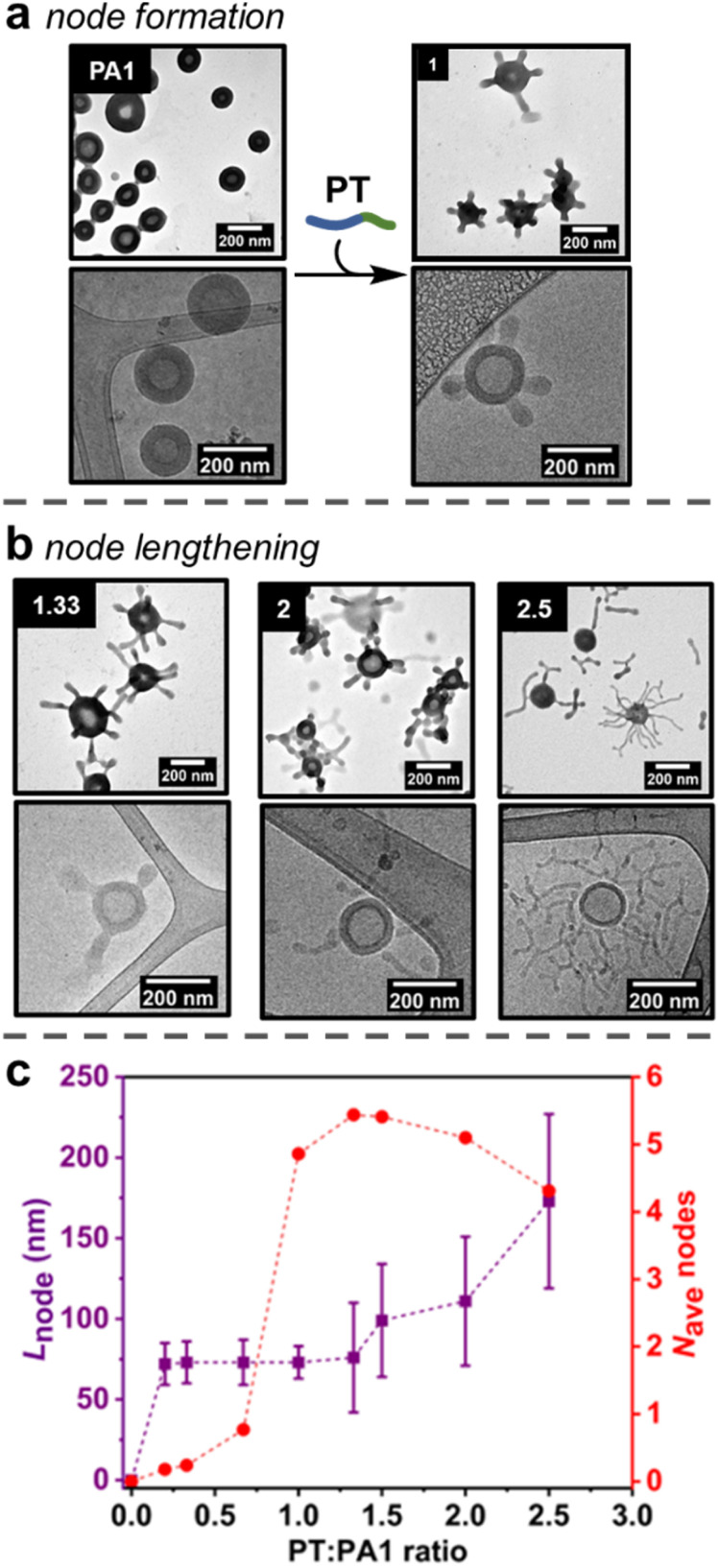
(a) Formation of nodes on outer surface of PA1 polymersomes, as shown by (dry-state and cryo-) TEM analysis. The equivalents of added PT chains *versus*PA1 chains is indicated in the top left of each image. (b) Lengthening of nodes on outer surface of PA1 polymersomes, as shown by TEM (dry-state and cryo-) analysis. (c) Evolution of node length (purple squares) and average number of nodes (red circles) per PA1 polymersome as a function of added PT. Error bars indicate standard deviation. Dry-state TEM samples were stained using 1 wt% uranyl acetate (UA) solution prior to imaging.

**Fig. 3:**
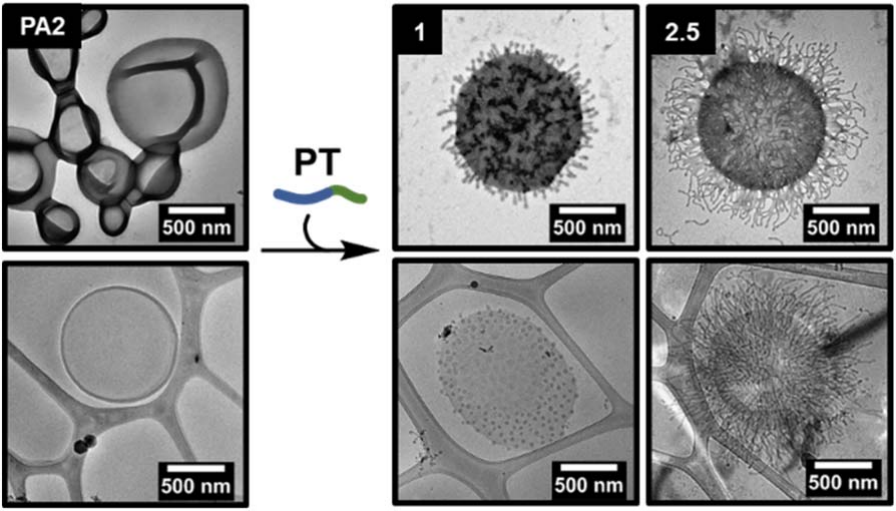
Formation and lengthening of nodes on outer surface of PA2 polymersomes, as shown by (dry-state and cryo-) TEM analysis. The equivalents of added PT chains *versus*PA2 chains is indicated in the top left of each image. Dry-state TEM samples were stained using 1 wt% uranyl acetate (UA) solution prior to imaging.

Polymersomes (Fig. 2a and 3) were analysed by Dynamic Light Scattering (DLS) and (cryogenic)-transmission electron microscopy ((cryo)-TEM). DLS analysis (ESI, Fig. S4†) showed both PISA formulations produced a monomodal population of polymersomes of relatively low polydispersity (hydrodynamic diameter, *D*_h_ = 193 ± 33 nm for PA1 and 867 ± 407 nm for PA2). TEM image analysis was used to measure mean particle diameter and membrane thickness in each case (ESI, Fig. S5†). Particle diameters, as measured by TEM, for both populations were similar (192 ± 38 nm for PA1 and 698 ± 338 nm for PA2) to those determined by DLS. The average membrane thickness, *M*_ave_, was approximately the same for both populations (*M*_ave_ = 42 ± 2 nm for PA1 and 44 ± 4 nm for PA2).

A shorter polymer, PT, containing complementary thymine side chains (Fig. 1c) was also synthesised *via* aqueous RAFT-PISA and characterised by ^1^H NMR and SEC analysis (ESI, Fig. S7 and S8†). The same length for the hydrophilic block as PA1/PA2 was targeted for PT, to ensure steric crowding at the hydrophobic/hydrophilic interface upon insertion of PT into the polymersome membrane. Self-assembly of pure PT during the PISA process in aqueous solution at [solids] = 5% w/w produced a mixture of spherical and worm-like micelles of uncontrolled length (ESI, Fig. S10 and S11†).

### Formation of nodes on the outer surface of PA1

Studies initially focussed on adding PT to the smaller PA1 particles, as quantification of formed nodes was possible by TEM analysis. First, increasing amounts of PT (0.20, 0.33, 0.67 or 1.00 equivalents of PT chains *versus*PA1 chains) in an aqueous stock solution (concentration = 5 mg mL^−1^) were added as single additions to aqueous solutions of PA1 (concentration = 0.5 mg mL^−1^). The PA1 polymersomes remained spherical after the addition of PT. However, local deformations – nodes – were observed on the outer membrane surface, as revealed by dry-state TEM analysis (Fig. 2a, ESI, Fig. S12†). Micellar PT was not observed by TEM, indicating the polymer was fully incorporated within the membrane of PA1. The length of nodes formed on PA1 was controlled by the amount of added PT. This indicates that PT particles, which form during RAFT-PISA with uncontrolled length, fully disassemble upon insertion into PA1, rather than attaching to the membrane surface.

Statistical analysis was performed on TEM images of PA1+ PT in order to further understand the formation of nodes. The average number of nodes per particle (Fig. 2c and ESI, Fig. S14†) increased dramatically when increasing the ratio of PT:PA1 from 0.67 (one node per particle) to 1.00 (five nodes per particle). Node length (Fig. 2c) and polymersome membrane thickness (ESI Fig. S15†) remained approximately constant over this range.

### Lengthening of nodes

The number of nodes per PA1 particle did not significantly change on addition of between 1.00 and 2.00 equivalents of PT (Fig. 2c). Instead, increasing the amount of added PT over this range caused pre-formed nodes to lengthen (Fig. 2b) to 111 ± 40 nm for 2.00 equivalents of added PT. Polymersome membrane thickness again remained constant over this range. Finally, addition of 2.50 equivalents of PT produced longer (173 ± 54 nm) nodes with some branching, as well as observation of a small number of nodes becoming detached from the PA1 polymersomes. These were observed as discrete ‘worm-like’ micelles by TEM. Detachment resulted in a slight decrease in average nodes per particle with 2.50 equivalents of added PT.

### Proposed mechanism of node formation and lengthening

The addition of PT caused changes to PA1 polymersomes in three steps: (1) nodes of near uniform length gradually form when less than 1.00 equivalent of PT is added; (2) upon addition of 1.00 equivalent of PT, the number of nodes increased rapidly to five nodes per particle; (3) after 1.50 or more equivalents of PT were added, the nodes grew longer but the overall number of nodes per particle remained approximately constant. These results cast light on the mechanism of node formation:

(1) A small number of nodes form when <1.00 equivalents of PT have been added, suggesting there needs to be an accumulation of steric crowding (*i.e.*, an increase in free energy due to strain) at the hydrophobic/hydrophilic interface before the formation of nodes becomes favourable.^48^

(2) Once this crowding reaches a critical threshold, nodes form rapidly. The uniformity (Fig. 2c) in length of newly formed nodes suggests that they represent a local minimum in free energy – the formation of additional nodes is initially favoured over node lengthening. Such node formation occurs similarly in lipid membranes.^49^ Mixing lipids that preferentially form disordered phases (such as phospholipids) with those that form ordered phases (rigid lipids, such as cholesterol) is energetically disfavoured. This is because polar headgroups come into contact with hydrophobic lipid tails and produce a force known as line tension.^50^ A similar effect occurs in PA1 membranes containing PT: as the two polymers have mismatched hydrophobic chain lengths, steric crowding occurs at the hydrophobic/hydrophilic interface that provides a driving force for node formation.^44^ In addition, no deformation of the internal membrane surface is observed, likely because PT cannot diffuse through the adenine-containing domains formed by PA1. As PT cannot penetrate throughout the polymersome membrane, the ‘area balance’ between the outer and inner membrane surfaces is perturbed.^51^ When this imbalance occurs in lipid membranes, stress can be released by echinocytosis (the generation of a spiky morphology), similar to node formation in this study.^52^ The ability to maintain an overall isotropic morphology whilst deforming the outer membrane surface is complementary to other recent reports, which describe the complete deformation of polymersome shape to produce anisotropic structures.^13–15^

Reduction of line tension in lipid membranes is achieved by coalescence of similar lipids to minimise the unfavourable interface. This can also serve to localise lipids with higher intrinsic curvature. Hydrogen bonded PT+PA1 have a higher intrinsic curvature than PA1 alone. This is because the volume ratio of the hydrophobic:hydrophilic domains is greater in PA1 than PT.^53^ Budding (*i.e.*, node formation) then occurs in lipids when sufficient excess free energy is present to overcome the membrane bending energy barrier.^54,55^ Therefore, budding observed here is driven by a free energy increase due to steric crowding. It is likely that PT (bonded to PA1) resides principally in and around the nodes, where local curvature is highest.

(3) Once the number of nodes per particle reaches a critical threshold (five per particle on average), further node formation becomes less favourable *versus* node lengthening, implying that further distortion of the outer membrane monolayer carries an increasingly large energetic penalty. Lengthening of nodes to form spherical and tubular structures also occurs when adding amphiphilic molecules to lipid membranes.^56^ When line tension is sufficient in lipid membranes, severing of the bud neck occurs, releasing an exovesicle without lysis of the parent membrane. Similar behaviour is observed with PA1, as some long nodes detach as cylindrical micelles once 2.50 equivalents of PT have been added. Finally, addition of an amphiphile with a comparatively large polar unit (such as PT) biases the formation of anisotropic, rather than spherical nodes.^56^

### Node growth on the surface of PA2

The addition of PT to PA2 polymersomes was then studied to determine whether the proposed node formation/lengthening mechanism was applicable to larger polymersomes (Fig. 3). As with PA1, varying amounts of an aqueous solution of PT were added to an aqueous solution of PA2. Quantitative analysis of the dry-state TEM images to determine the average number of nodes per particle was not possible as larger polymersomes obscured some nodes, however the average length of nodes could be measured (ESI, Fig. S13 and S16†). A markedly higher number of nodes per particle were formed in comparison to PA1 and these were packed more densely on the polymersome surface. The greater number of nodes was attributed firstly to the larger accessible surface area (approximately 13× greater for PA2 than PA1, based on average particle diameters measured by TEM). Also, membranes of PA2 polymersomes have a lower intrinsic curvature than for PA1. This results in a lower bending resistance.^57,58^ We propose this permits denser coverage with nodes. Addition of increasing amounts of PT also caused a lengthening of nodes, as for PA1.

### Control experiments

**Fig. 4:**
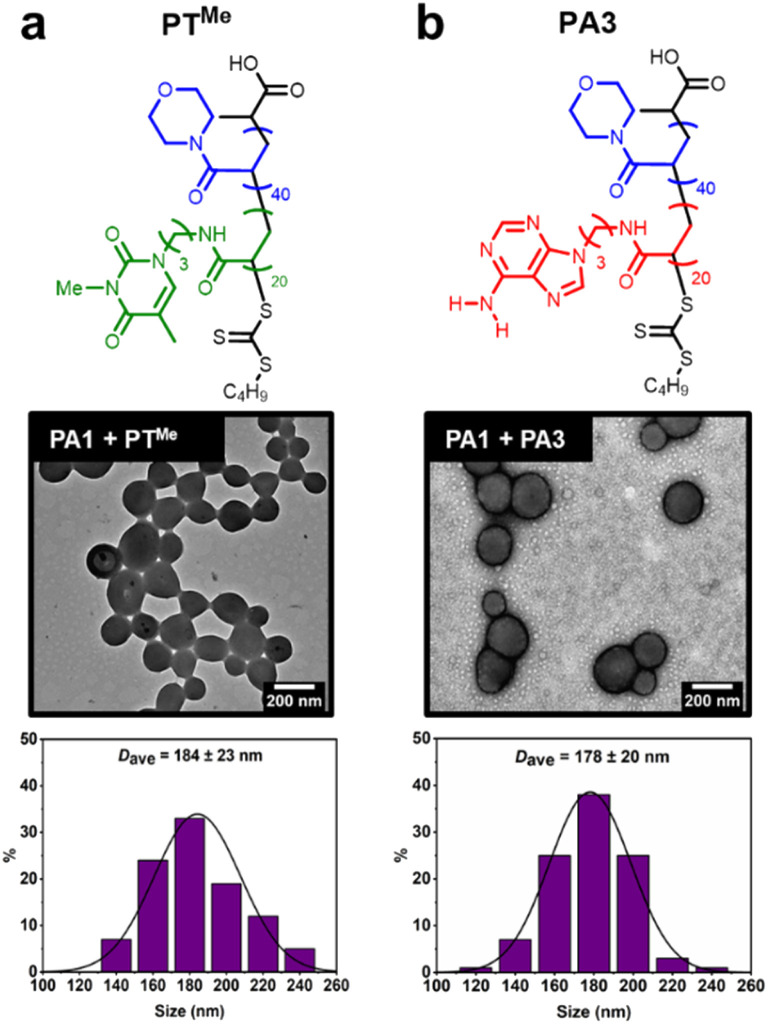
Attempted node formation by mixing control polymers (a) PT^Me^ or (b) PA3 with PA1 polymersomes. Histograms plotted by measuring diameter of >100 polymersome particles. Dry-state TEM samples were stained using 1 wt% uranyl acetate (UA) solution prior to imaging.

Our previous study demonstrated that the formation of hydrogen bonds between complementary nucleobases provides the essential driving force for morphological transformations.^44^ To confirm whether this was also a requirement with node formation on polymersomes, control experiments were performed using two polymers analogous to PT: PT^Me^, a methylated analogue where each thymine unit is unable to donate a hydrogen bond, and PA3, where thymine side chains have been replaced with adenine ones (Fig. 4).

These polymers were also synthesised by aqueous RAFT-PISA (see ESI†). Self-assembly of PT^Me^ and PA3 was observed by DLS (ESI, Fig. S21†), whilst nano-objects could not be observed by TEM for PT^Me^. TEM imaging of PA3 showed the presence of spherical and worm-like micelles (ESI, Fig. S22†).

The ability of PT^Me^ and PA3 to produce nodes on the surface of PA1 polymersomes was investigated. One equivalent (sufficient to cover particles in nodes when using PT) of each polymer was added to separate solutions of PA1 polymersomes. No node formation was observed in either case, indicating that complementary nucleobase pairing is required for such a process. PA1 polymersomes remained approximately the same size, suggesting that PT^Me^ and PA3 also did not insert and cause particle swelling. TEM imaging of PA1 mixed with PA3 shows the presence of both polymersomes and spherical micelles with similar dimensions to those seen in unmixed samples (Fig. 4b). Therefore, due to the lack of complementary nucleobase pairing, PT^Me^ and PA3 can neither facilitate node growth, nor do they insert into PA1 polymersomes. In other words, insertion into PA1 is selective for PT. Colocalisation of the two polymers upon node formation was confirmed using confocal microscopy to image a sample of PA2 labelled with BODIPY-FL (a green emitter) and PT labelled with BODPI 630/650 (a red emitter) (ESI, Fig. S26†).

## Conclusions

This study demonstrates the use of complementary nucleobase pairing to drive the formation and lengthening of nodes on the outer surface of polymersomes. Node growth and length are determined by the ratio of complementary nucleobases, thymine:adenine, within the polymersome membrane. This is controlled by adding a specified amount of polymer to tailor the nucleobase ratio. The proposed mechanism of node formation and lengthening is reminiscent of biological exovesicle formation; membrane deformation results due to the interaction between two components possessing different intrinsic curvature.^59^

This mechanism has given access to two different types of assemblies depending on the size of initial polymersome seed. When using a smaller seed, patchy particles with a few tentacle-like protrusions are produced. Nanoparticles coated with such nodes have been shown to have superior plasmonic properties to other morphologies,^60,61^ but controlling their length and number has proved elusive.^62–66^

Using larger polymersome seeds gives access to assemblies that resemble ‘hairy’ nanoparticles, which are usually formed by decorating a core with outward dangling polymer chains.^67^ Adding ‘hairs’ to nanoparticles can produce favourable properties, such as higher viscosity,^68,69^ greater ability to separate gases^70^ and improved ionic conductivity.^71^ These particles are normally synthesised as composite materials using either a grafting-to (attaching preformed polymer chains) or grafting-from (initiated polymerisation from the surface) approach. This study provides access to a complementary method, with hairs (elongated nodes) and the ‘core’ both being formed of similar polymers. This ability to control the number and length of nodes on the surface of polymersomes using nucleobase pairing therefore may prove to be an enabling method for accessing the next generation of functional nanomaterials with precision.

## Data availability

The data that support the findings of this study are available in the ESI.†

## Author contributions

Conceptualisation: MT, SV, TRW, ROR. Experimental data collection: MT, SV, TRW, SDPF. Data analysis: MT, SDPF, ROR. Writing: MT, SDPF, ROR.

## Conflicts of interest

There are no conflicts to declare.

## Supplementary Material

SC-015-D3SC05915D-s001
