# Supplementary material for: Controlled node growth on the surface of polymersomes
Source: Chem Sci. 2024 Feb 16;15(12):4396–402. doi: 10.1039/d3sc05915d (PMC10952076; doi:10.1039/d3sc05915d)
Supplement: SC-015-D3SC05915D-s001 [file SC-015-D3SC05915D-s001.pdf]

Electronic Supplementary Material (ESI) for Chemical Science.
This journal is © The Royal Society of Chemistry 2024

Supplementary Information

"Controlled node growth on the surface of polymersomes"

Marjolaine Thomas, Spyridon Varlas, Thomas R. Wilks, Stephen D. P. Fielden and Rachel K. O'Reilly**

School of Chemistry, University of Birmingham, Edgbaston, Birmingham, B15 2TT, UK

*Corresponding Authors: s.fielden@bham.ac.uk, r.oreilly@bham.ac.uk

Contents

Experimental Section 2
Materials and Methods 2
Materials 2
Characterization techniques 2
Synthetic Methods 5
Synthesis of monomers AAm, TAm and T^{Me}Am 5
Synthesis of 2-(((butylthio)carbonothiolyl)thio)propanoic acid (CTA) 5
Synthesis of PNAM$_{40}$ (macro-CTA) 6
Synthesis of PNAM$_{40}$-*b*-PAAm$_x$ diblock copolymer nano-objects (**PA$_x$**) 7
Synthesis of PNAM$_{40}$-*b*-PTAm$_{20}$ diblock copolymer nano-objects (**PT**) 8
Supplementary Characterization Data for PNAM$_{40}$ mCTA 9
Supplementary Characterization Data for PNAM$_{40}$-*b*-PAAm$_x$ (**PA1** and **PA2**) 10
Supplementary Characterization for PNAM$_{40}$-*b*-PTAm$_{20}$ (**PT**) 15
Addition of **PT** to **PA1** and **PA2** Polymersomes 18
Synthesis and Characterization of PNAM$_{40}$-*b*-PT^{Me}Am$_{20}$ (**PT^{Me}$_{20}$**) and PNAM$_{40}$-*b*-PAAm$_{20}$ (**PA3**) 24
Supplementary Characterization Data for Morphological Transformation Control Experiments: Addition of non-complementary block copolymers to a **PA1** solution. 31
References 32

Experimental Section

Materials and Methods

Materials

2,2'-Azobis(isobutyronitrile) (AIBN) was obtained from Molekula and recrystallized from methanol. 2,2'-Azobis[2-(2-imidazolin-2-yl)propane]di-hydrochloride (VA-044) was purchased from Wako and was used without further purification. Adenine, sodium hydride (60% dispersion in mineral oil), acryloyl chloride, pyridine and 4-acryloylmorpholine (NAM) were purchased from Sigma-Aldrich and used without further purification. Triethylamine, DMF and DMSO were purchased from Fisher Scientific and used without further purification. Dialysis membranes (MWCO = 3.5 kDa) were purchased from Spectra/Por. Dry solvents (DMF and dioxane) were obtained by passing over a column of activated alumina using an Innovative Technologies solvent purification system.

Characterization techniques

^{1}H Nuclear Magnetic Resonance (NMR) Spectroscopy. Spectra were recorded at room temperature on a Bruker DPX-300, DPX-400 or HD500 spectrometer with D_2O, DMSO-d_6 or $CDCl_3$ as the solvent. Chemical shifts are quoted as δ in parts per million.

High-Resolution Mass Spectrometry (HRMS). Spectra were recorded by the MS Analytical Facility Service at the University of Birmingham on a Waters Xevo G2-XS Quadrupole Time-of-Flight mass spectrometer.

Size Exclusion Chromatography (SEC). Analysis was performed on an Agilent 1260 Infinity II LC system equipped with a Wyatt Optilab T-rEX differential refractive index detector (RI), an Agilent guard column (PLGel 5 μM, 50 × 7.5 mm) and two Agilent Mixed-C columns (PLGel 5 μM, 300 × 7.5 mm). The mobile phase was DMSO containing 0.1% w/w of LiBr or DMF

containing 5 mM NH_4BF_4 and was pumped at 50 °C and a flow rate of 1.0 mL min^{-1}. Detection was performed using an ultraviolet (UV) detector set to λ_{mCTA} = 309 nm. Number-average molecular weights (M_n), weight-average molecular weights (M_w) and dispersities ($Đ_M = M_w/M_n$) were determined using the Agilent GPC/SEC software (vA.02.01) against a 12-point calibration curve (550–2,210,000 g mol^{-1}) based on poly(methyl methacrylate) standards (Easivial PM, Agilent).

Differential Scanning Calorimetry (DSC). Determination of the glass transition temperature (T_g) for PNAM$_{40}$-*b*-PAAm$_{200}$ (**PA1**) and PNAM$_{40}$-*b*-PTAm$_{20}$ (**PT**) diblock copolymers was performed using a Mettler Toledo DSC 3 differential scanning calorimeter by heating the sample from 15 °C to 150 °C at a rate of 10 °C min^{-1} for two heating/cooling cycles. The T_g was determined from the inflection point in the second heating cycle of DSC. Collected data were processed using STARe software.

Dynamic Light Scattering (DLS). Hydrodynamic diameters (D_h) and size distributions of self-assembled nano-objects were determined using a Malvern Zetasizer NanoZS instrument operating at 25 °C with a 4 mW He-Ne 633 nm laser module. Measurements were made at a detection angle of 173° (back scattering). Four runs were obtained for each sample and Malvern DTS 6.20 software was used to analyse the data.

Transmission Electron Microscopy (TEM). Dry-state TEM imaging was performed on a JEOL JEM 1400 electron microscope at an acceleration voltage of 80 kV. All TEM samples were prepared on formvar carbon-coated copper grids (EM Resolutions). After 200-fold dilution with deionized water, a drop of sample (8 μL) was pipetted onto a grid and left for approximately one minute. Excess sample was blotted with filter paper and the grid was stained with 1 wt% uranyl acetate (UA) aqueous solution (8 μL) for one minute prior to blotting, drying and analysis. TEM images were analysed using the ImageJ software; at least 100 particles were

measured for each sample to obtain the number-average diameter, D_{ave} or the membrane thickness, M_{ave}.

Cryogenic Transmission Electron Microscopy (Cryo-TEM). Imaging was performed on a JEOL JEM-2100 Plus microscope operating at an acceleration voltage of 200 kV. Samples for cryo-TEM were prepared on lacey carbon-coated copper grids (EM Resolutions). After 200-fold dilution with deionized water, a drop of sample (8 μL) was pipetted onto the grid. The grid was blotted for five seconds and vitrified by being plunged into a pool of liquid ethane. The grids were transferred into a pre-cooled cryo-TEM holder using liquid nitrogen prior to microscopic analysis. Cryo-TEM images were analysed using the ImageJ software and at least 100 particles were measured for each sample to obtain number-average diameter, D_{ave}.

Confocal Laser Scanning Microscopy. Samples were prepared at 100-fold dilution and deposited on a glass slide. Images were acquired using an using Olympus FV3000 confocal laser Scanning Microscope and ImageJ image processing software after evaporation of the solvent. Assemblies tagged with BODIPY-FL amine dye (green) were excited using a 488 nm laser, while assemblies tagged with BODIPY 630/650 amine dye (red) were excited using a 633 nm laser. Both channels were used at the same time to produce overlays.

Synthetic Methods

Synthesis of monomers AAm, TAm and T^{Me}Am

3-(Adenine-9-yl)propyl acrylamide (**AAm**), 3-(thymin-1-yl)propyl acrylamide (**TAm**) and 3-(3-methylthymin-1-yl)propyl acrylamide (**T^{Me}Am**) monomers were synthesized using procedures reported in the literature.[1]

Synthesis of 2-(((butylthio)carbonothiolyl)thio)propanoic acid (CTA)

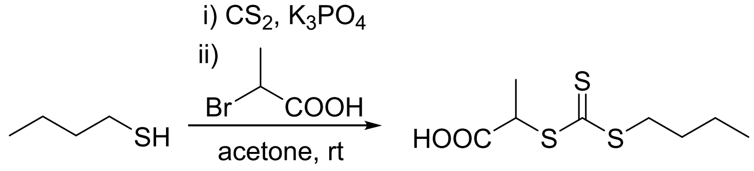

Scheme S1: Synthesis of **CTA**

Adapted from a literature procedure.[1] K_3PO_4 (20.9g, 99 mmol, 1.0 eq) and butanethiol (10.7 mL, 99 mmol, 1.0 eq) were dissolved in 100 mL of acetone. An excess of carbon dislufide (CS_2) (8.93 mL, 149 mmol, 1.5 eq) was added and stirred for 0.5 h. 2-Bromopropanoic acid (8.91 mL, 99 mmol, 1.0 eq) was added and precipitation was observed. The reaction mixture was stirred for 2 h. The solution was then passed through a filter paper and concentrated under reduced pressure. 1M HCl (50 mL) was added and the solution was extracted with EtOAc (2 x 100 mL). The combined extracts were washed with brine (2 x 100mL) and dried over $MgSO_4$. The solvent was removed under reduced pressure. The product was then recrystallized from hexane to afford a yellow powder (5.6 g, 23%). ^{1}H NMR (400 MHz, $CDCl_3$) δ: 9.82 (br, 1H, COO***H***), 4.87 (q, J = 10.0 Hz, 1H, ***H***C-CO), 3.38 (t, J = 10.0 Hz, 2H, SC(=S)-C***H***$_2$), 1.70 (m, 2H, SC(=S)-C-C***H***$_2$), 1.62 (d, J = 10.0 Hz, 3H, OC-C-C***H***$_3$), 1.42 (m, 2H, SC(=S)-C-C-C***H***$_2$), 0.94 (t, J = 10.0 Hz, 3H, CH_2C***H***$_3$); ^{13}C NMR (400 MHz, $CDCl_3$) δ: 177.4, 165.2, 47.7, 37.3, 30.1, 22.3, 16.8, 13.8 ppm; HRMS (m/z) found 261.0055, calc. 261.0054 $[M+Na]^+$.

Synthesis of $PNAM_{40}$ (macro-CTA)

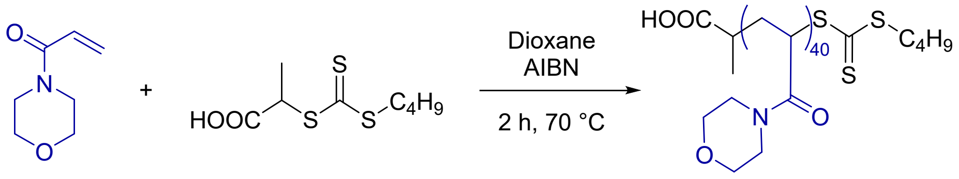

Scheme S2: Synthesis of $PNAM_{40}$ macro-CTA.

Adapted from a literature procedure.[1] A 10 mL ampoule was charged with NAM (4-acryolylmorpholine, 126 µL, 1.0 mmol, 100 eq), 2-(((butylthio)carbonothiolyl)thio)-propanoic acid (2.4 mg, 0.01mmol, 1 eq), AIBN (1.025 mg, 0.01 mmol, 1.0 eq) and 0.5 mL of dioxane. The mixture was thoroughly degassed via four freeze-pump-thaw cycles, filled with nitrogen gas and then immersed in an oil bath at 70 °C for 2 h. The solution was then diluted in water (2 mL) and dialysed against water, incorporating at least six water changes. Water was removed by freeze-drying to afford a light-yellow solid.

Synthesis of PNAM$_{40}$-*b*-PAAm$_x$ diblock copolymer nano-objects (PA)

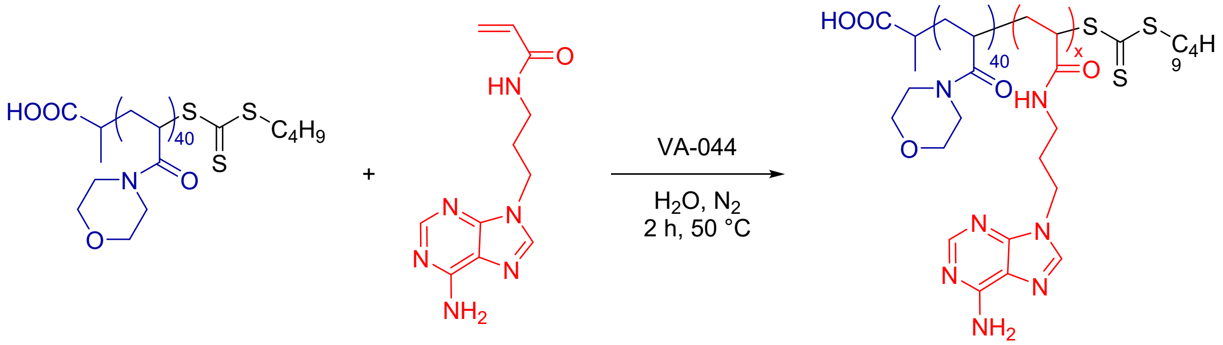

Scheme S3: Synthesis of **PNAM$_{40}$-*b*-PAAm$_x$** diblock copolymer nano-objects (**PA**) via aqueous RAFT-mediated PISA.

A typical synthetic procedure to synthesise PNAM$_{40}$-*b*-PAAm$_{200}$ (**PA1**) diblock copolymer vesicles at [solids] = 10% w/w *via* aqueous RAFT-mediated PISA is described. PNAM$_{40}$ macro-CTA (9.0 mg, 1.59×10^{-6} mol, 1.0 eq.), AAm (80 mg, 3.17×10^{-4} mol, 200 eq.) and VA-044 (0.10 mg, 3.17×10^{-7} mol, 0.2 eq.) (52 µL of a stock solution of 2.0 mg of VA-044 in 1.0 mL of water) were dispersed in deionized (DI) water (0.89 mL) and sealed in a 7 mL vial containing a magnetic stirrer bar. The resulting monomer-in-water solution was degassed by sparging with nitrogen gas for 15 min. The sealed vial was heated at 50 °C with magnetic stirring for 2 h to ensure full monomer conversion. After this period, the reaction mixture was exposed to air and allowed to cool to room temperature. **PA2** polymersomes were formed at [solids] = 15% w/w. The **PA2** solution was further dialyzed to remove any unreacted monomer. DLS analysis and dry-state/cryo-TEM imaging were performed on samples after dilution to an appropriate analysis concentration.

Synthesis of PNAM$_{40}$-*b*-PTAm$_{20,}$ diblock copolymer nano-objects (PT)

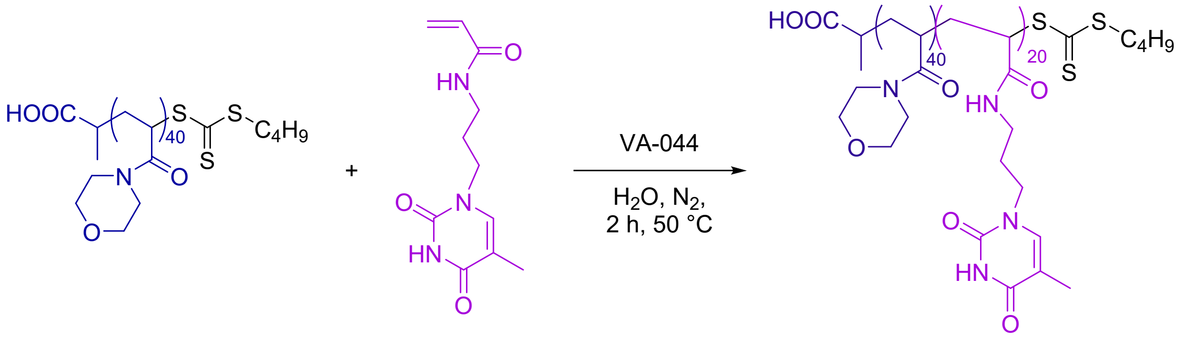

S

cheme S4: Synthesis of PNAM$_{40}$-*b*-PTAm$_{20}$ (**PT**) diblock copolymer nano-objects by aqueous RAFT-mediated PISA.

A typical synthetic procedure to synthesise PNAM$_{40}$-*b*-PTAm$_{20}$ (**PT**) diblock copolymer nano-objects at [solids] = 5% w/w *via* aqueous RAFT-mediated PISA is described. PNAM$_{40}$ macro-CTA (24 mg, 4.27×10^{-6} mol, 1 eq.), AAm (20 mg, 8.55×10^{-5} mol, 20 eq.) and VA-044 (0.27 mg, 8.55×10^{-7} mol, 0.2 eq.) (68 µL of a stock solution of 2 mg of VA-044 in 1 mL of water) were dispersed in deionized (DI) water (0.88 mL) and sealed in a 7 mL vial containing a magnetic stirrer bar. The resulting monomer-in-water solution was degassed by sparging with nitrogen gas for 15 min. The sealed vial was heated at 50 °C with magnetic stirring for 2 h to ensure full monomer conversion. After this period, the reaction mixture was exposed to air and allowed to cool to room temperature. DLS analysis and dry-state TEM imaging were performed on samples after dilution to an appropriate analysis concentration.

Supplementary Characterization Data for PNAM$_{40}$ mCTA

Table S1: Molecular characteristics of PNAM$_{40}$ mCTA prepared via RAFT solution polymerization in dioxane, as determined by ^{1}H-NMR spectroscopy and SEC analysis.

Polymer	Targeted DP	% Conv.[a]	$M_{n, NMR}$[b] / kDa	$M_{n, SEC}$[c] / kDa	$Đ_{M, SEC}$[c]
PNAM$_{40}$ mCTA	40	> 99	5.6	8.1	1.13

[a]Monomer conversion calculated from ^{1}H-NMR spectroscopy in D_2O. [b]Calculated from conversion. [c]M_n and $Đ_M$ values calculated from PMMA standards using DMSO + 0.1% w/w LiBr as the eluent.

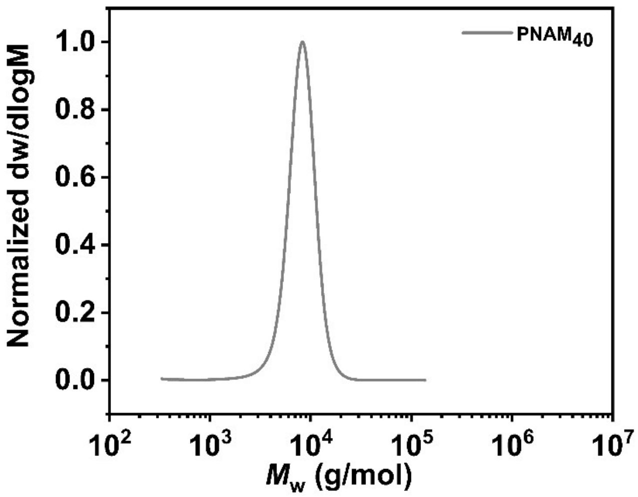

Figure S1: Normalized molecular weight distribution trace of PNAM$_{40}$ mCTA (DMSO + 0.1% w/w LiBr as eluent, PMMA standards).

Supplementary Characterization Data for PNAM$_{40}$-*b*-PAAm$_x$ (PA1 and PA2)

Table S2: Molecular characteristics of PNAM$_{40}$-*b*-PAAm$_{200}$ (**PA1**) and PNAM$_{40}$-*b*-PAAm$_{216}$ (**PA2**) prepared via aqueous RAFT-mediated PISA, as determined by ^{1}H-NMR spectroscopy and SEC analysis.

Polymer	Targeted DP	% Conv.[a]	$M_{n,\,NMR}$[b] / kDa	$M_{n,\,SEC}$[c] / kDa	$Đ_{M,SEC}$[c]
**PA1**	200	> 99	56.1	77.9	1.59
**PA2**	300	72	60.1	92.0	3.05

[a]Monomer conversion calculated from ^{1}H-NMR spectroscopy in D_2O. [b]Calculated from conversion. [c]M_n and $Đ_M$ values calculated from PMMA standards using DMSO + 0.1% w/w LiBr as the eluent.

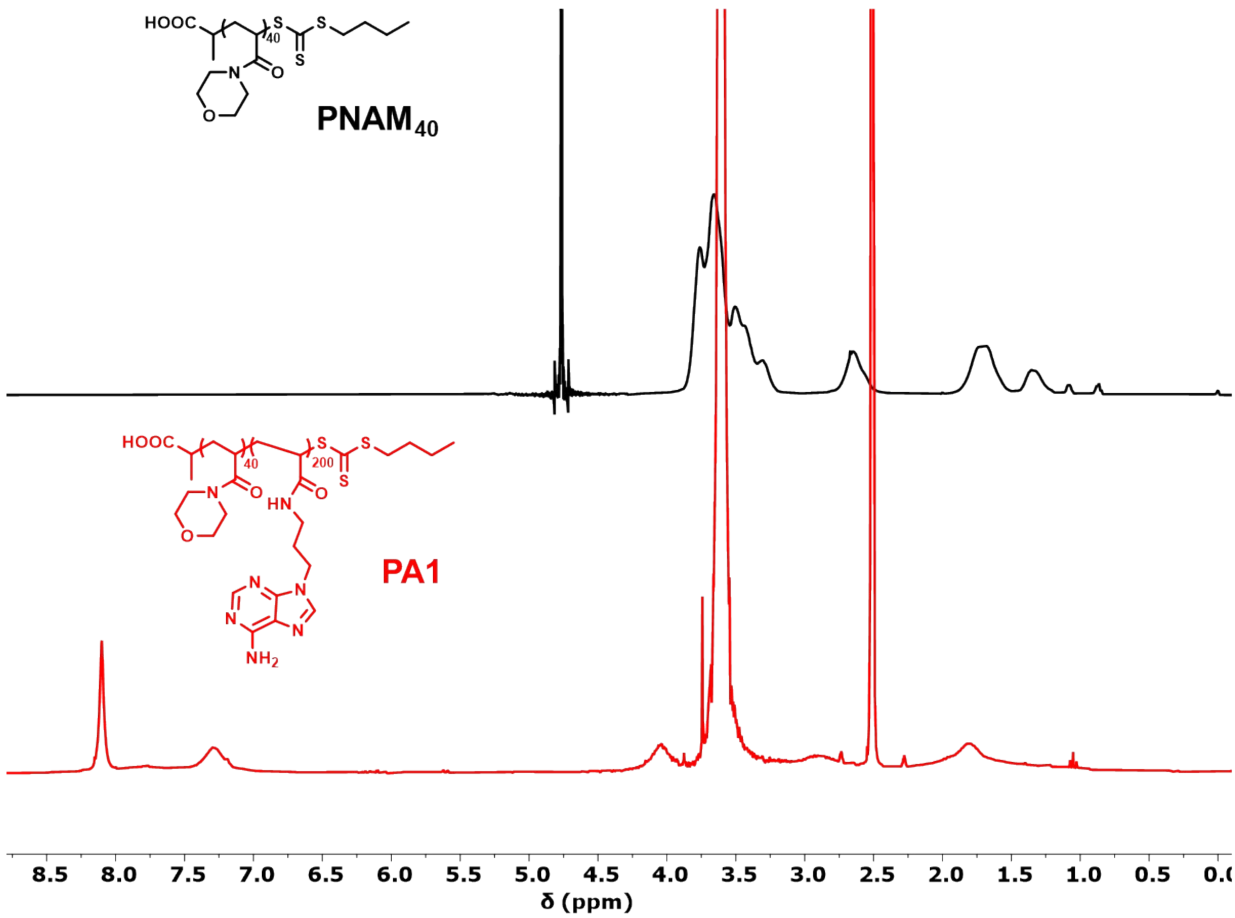

Figure S2: ^{1}H-NMR spectra of $PNAM_{40}$ macro-CTA in D_2O and crude $PNAM_{40}$-*b*-$PAAm_{200}$ (**PA1**) in DMSO-d_6 (400 MHz).

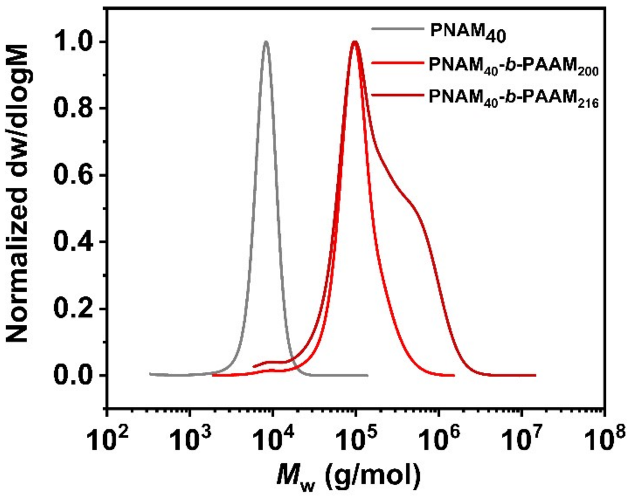

Figure S3: Normalized molecular weight distributions of $PNAM_{40}$, **PA1** and **PA2** (DMSO + 0.1% w/w LiBr as eluent, PMMA standards).

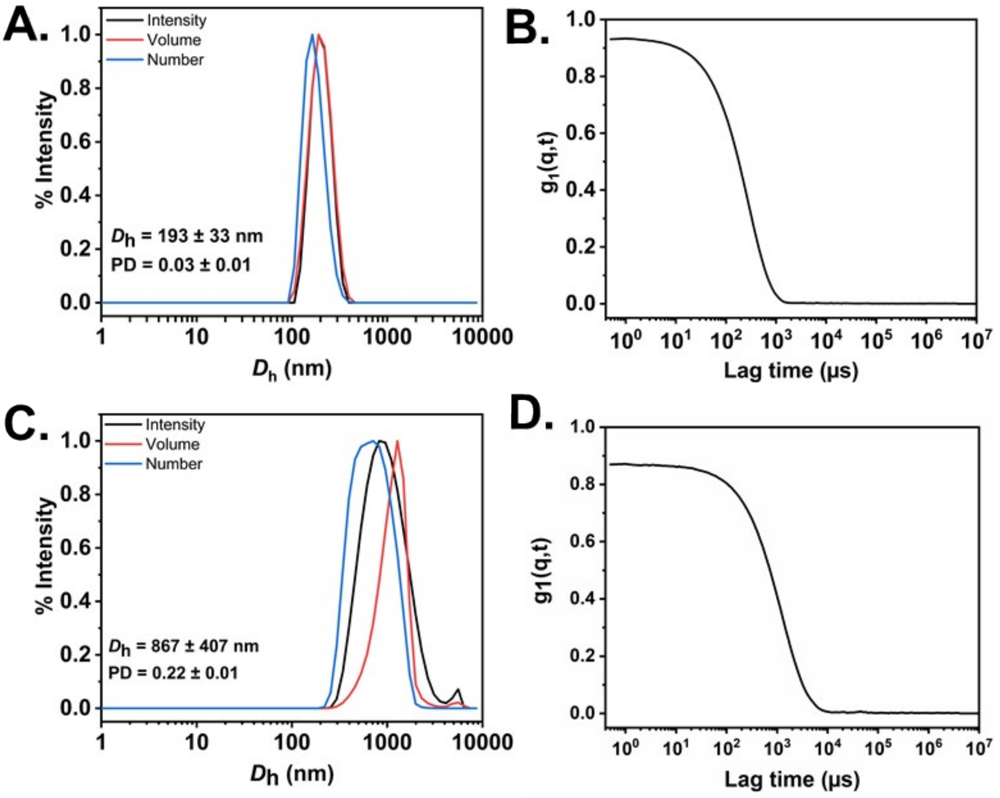

Figure S4: DLS particle size distributions and corresponding correlogram graph results of **PA1** (A and B) and **PA2** (C and D).

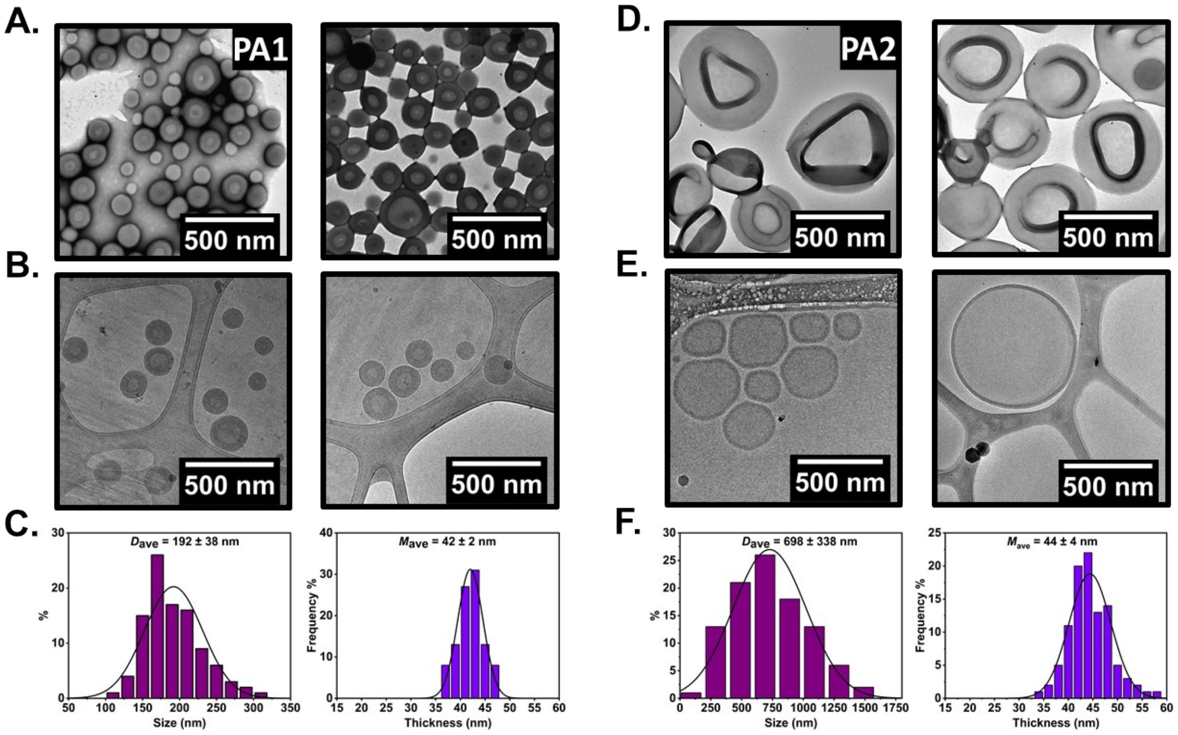

Figure S5: (A) and (D) dry-state TEM images of PNAM$_{40}$-*b*-PAAm$_{200}$ (**PA1**) and PNAM$_{40}$-*b*-PAAm$_{216}$ (**PA2**) diblock copolymer vesicles, respectively. (B) and (E) cryo-TEM images of PNAM$_{40}$-*b*-PAAm$_{200}$ (**PA1**) and PNAM$_{40}$-*b*-PAAm$_{216}$ (**PA2**) diblock copolymer vesicles. (C) and (F) histograms of **PA1** and **PA2** diblock copolymer vesicles' average diameter, D_{ave}, and average membrane thickness, M_{ave}, values calculated from analysis of TEM images. Dry-state samples were stained using 1 wt % uranyl acetate (UA) solution.

Table S3: Summary of DLS and TEM data of **PA1** and **PA2** block copolymer vesicles obtained by aqueous RAFT-mediated PISA.

**Particle**	D_h **(nm)**[a]	**PD**[a]	D_{ave} **(nm)**[b]	M_{ave} **(nm)**[b]	**Morphology**[c]
**PA1**	193 ± 33	0.03 ± 0.01	192 ± 38	42 ± 2	P
**PA2**	867 ± 407	0.22 ± 0.01	698 ± 338	44 ± 4	P

[a]D_h and PD values measured from DLS analysis (the error shows the standard deviation from 3 repeat measurements). [b]D_{ave} values were obtained from TEM analysis. [c]Morphologies observed from dry-state TEM imaging, using 1 wt % uranyl acetate (UA) solution for staining (Key: P – polymersomes).

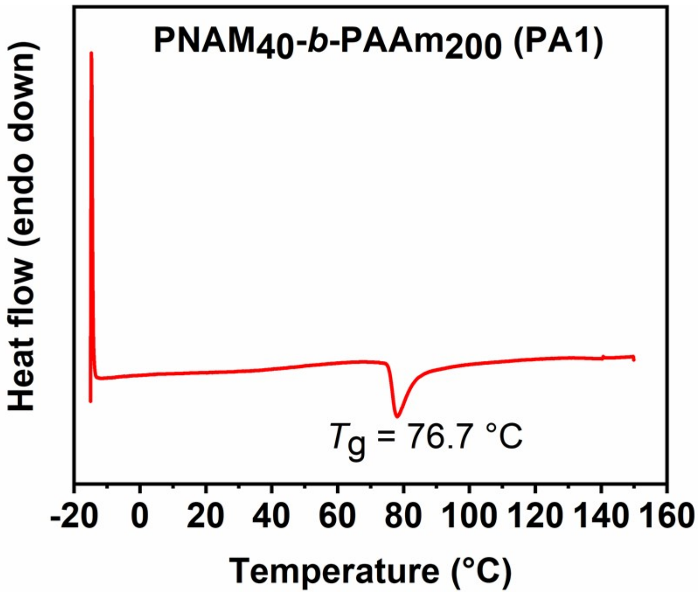

Figure S6: DSC thermogram of $PNAM_{40}$-*b*-$PAAm_{200}$ (**PA1**) (heating rate 10 °C/min). The endothermic peak observed at 76.7 °C corresponds to the T_g of the polymer.

Supplementary Characterization for PNAM$_{40}$-*b*-PTAm$_{20}$ (PT)

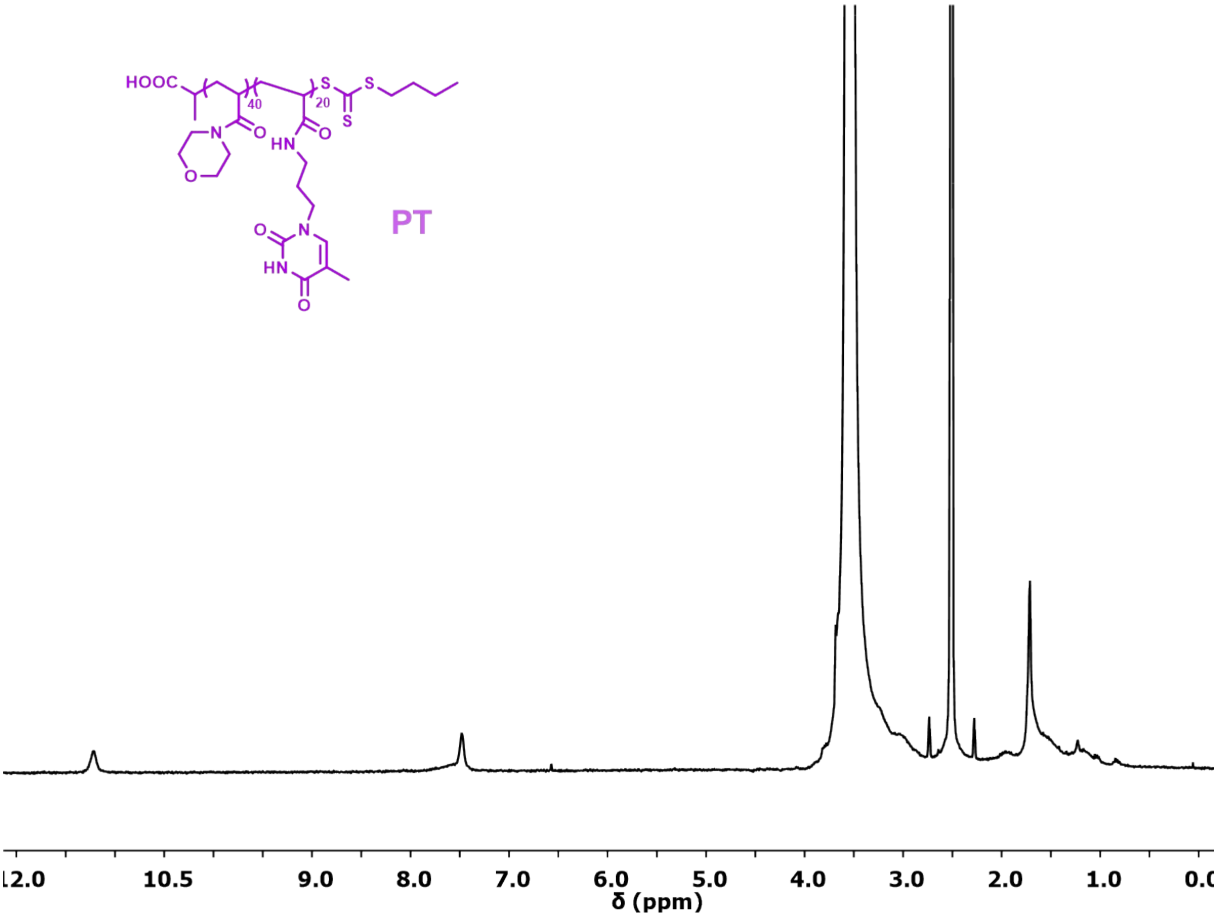

Figure S7: ^{1}H-NMR spectra of crude PNAM$_{40}$-*b*-PTAm$_{20}$ (**PT**) obtained by aqueous RAFT-mediated PISA recorded in DMSO-d_6 (400 MHz).

Table S4: Molecular characteristics of PNAM$_{40}$-*b*-PTAm$_{20}$ (**PT**) prepared via aqueous RAFT-mediated PISA, as determined by ^{1}H-NMR spectroscopy and SEC analysis.

Polymer	% Conv.[a]	$M_{n, NMR}$[b] / kDa	$M_{n, SEC}$[c] / kDa	$Đ_{M, SEC}$[c]
**PT**	> 99	10.3	16.6	1.18

[a]Monomer conversion calculated from ^{1}H-NMR spectroscopy in D_2O. [b]Calculated from conversion. [c]M_n and $Đ_M$ values calculated from PMMA standards using DMSO + 0.1% w/w LiBr as the eluent.

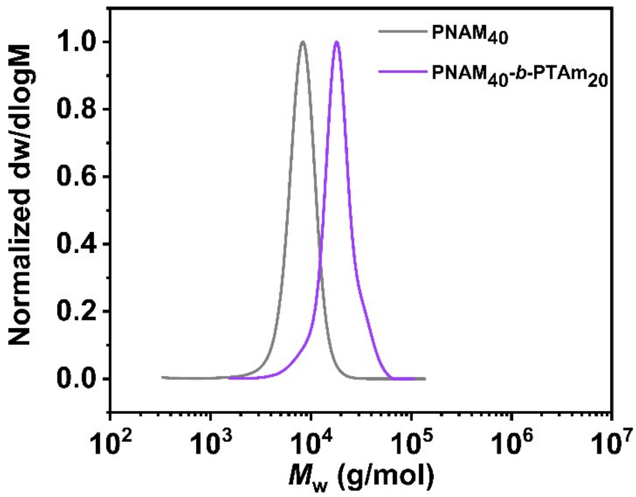

Figure S8: Normalized molecular weight distributions of $PNAM_{40}$ mCTA and $PNAM_{40}$-*b*-$PTAm_{20}$ (**PT**) (DMSO + 0.1% w/w LiBr as eluent, PMMA standards).

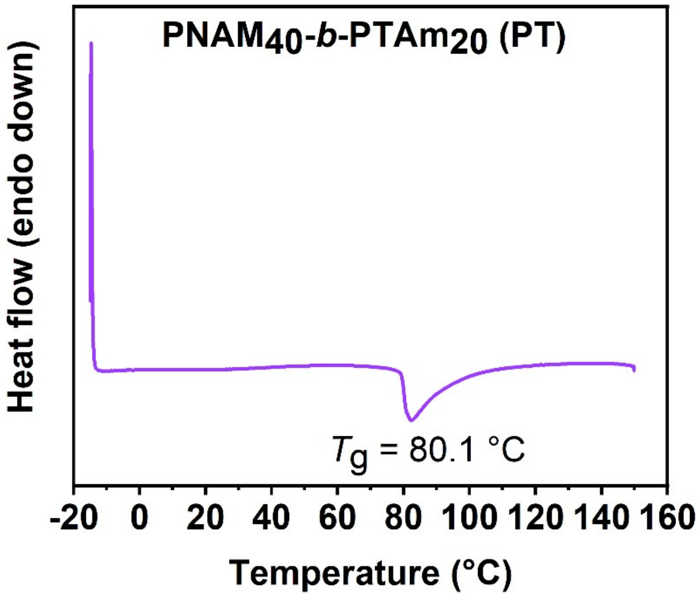

Figure S9: DSC thermogram of $PNAM_{40}$-*b*-$PTAm_{20}$ (**PT**) (heating rate 10 ^{o}C/min). The endothermic peak observed at 80.1 °C corresponds to the T_g of the polymer.

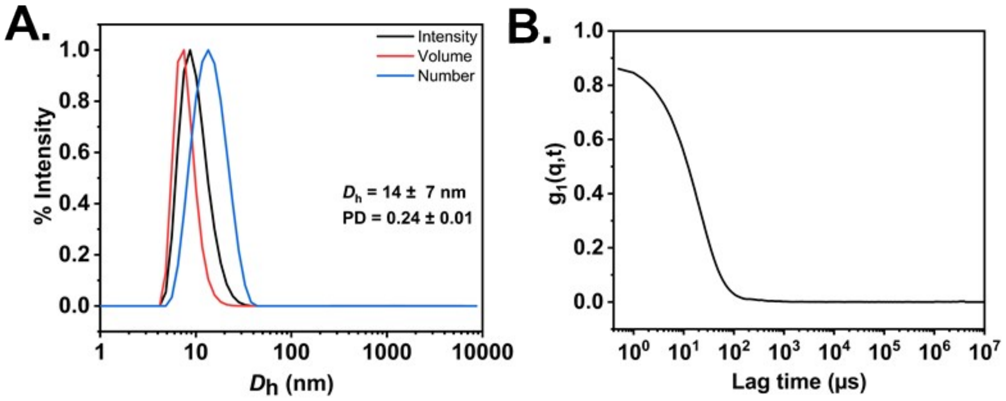

Figure S10: DLS particle size distribution and corresponding correlogram graph results of **PT**.

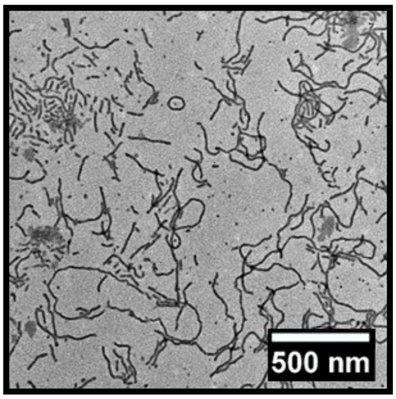

Figure S11: TEM image of **PT** ($PNAM_{40}$-*b*-$PTAm_{20}$) diblock copolymer nano-objects. The sample was stained with 1 wt % uranyl acetate (UA) solution.

Addition of PT to PA1 and PA2 Polymersomes

Initial solutions of **PA1** and **PA2** were prepared at a concentration of 0.5 mg mL^{-1} in water, whilst **PT** was diluted with water to 5 mg mL^{-1}. **PT** was added to **PA1** or **PA2** in different ratios and stirred for 2 h.

Table S5: Volumes of solutions **PA1** and **PT** required for mixing at different ratios.

PT:PA1 ratio	V_{PA1} (mL)	V_{PT} (µL)
0.2	1	3.6
0.33	1	5.5
0.67	1	12.8
1	1	18.3
1.33	1	24.4
1.5	1	27.3
2	1	36.7
2.5	1	45.5

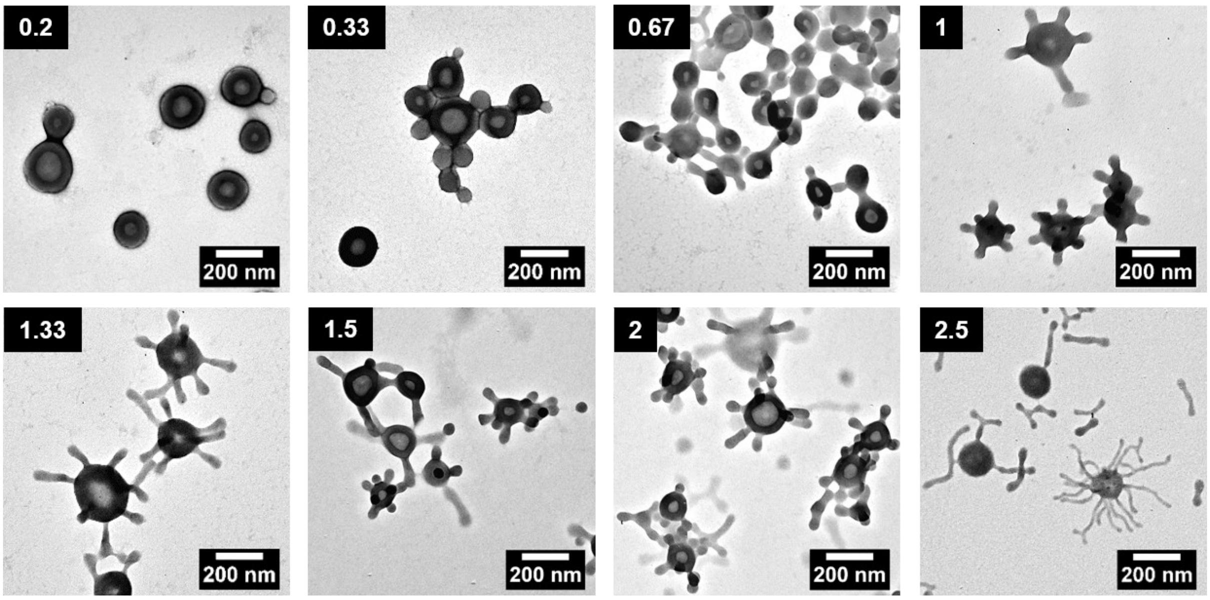

Figure S12: Additional dry-state TEM images of the particles formed by the addition of **PT** to **PA1**. The number of equivalents of added **PT** is indicated in the top left corner of each image.

Table S6: Volumes of solutions **PA2** and **PT** required for mixing at different ratios.

**PT:PA2 ratio**	**V_{PA2} (mL)**	**V_{PT} (μL)**
0.2	1	3.4
0.33	1	5.6
0.67	1	11.3
1	1	16.9
1.33	1	22.5
1.5	1	25.4
2	1	33.9
2.5	1	42.4

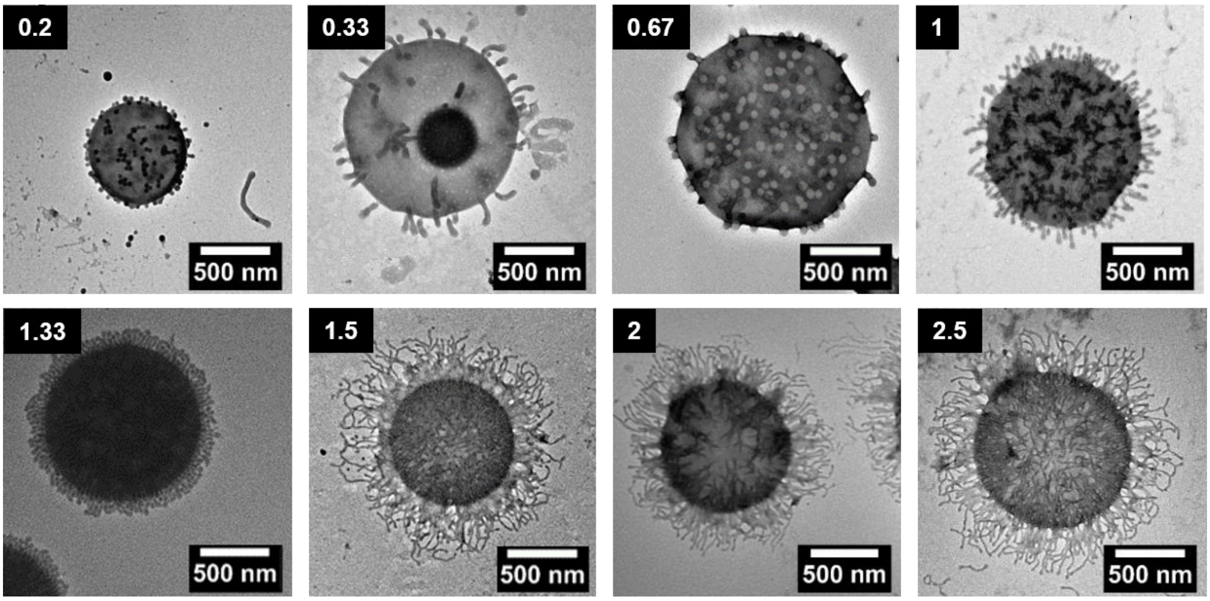

Figure S13: Additional dry-state TEM images of the particles formed by the addition of **PT** to **PA2**. The number of equivalents of added **PT** is indicated in the top left corner of each image.

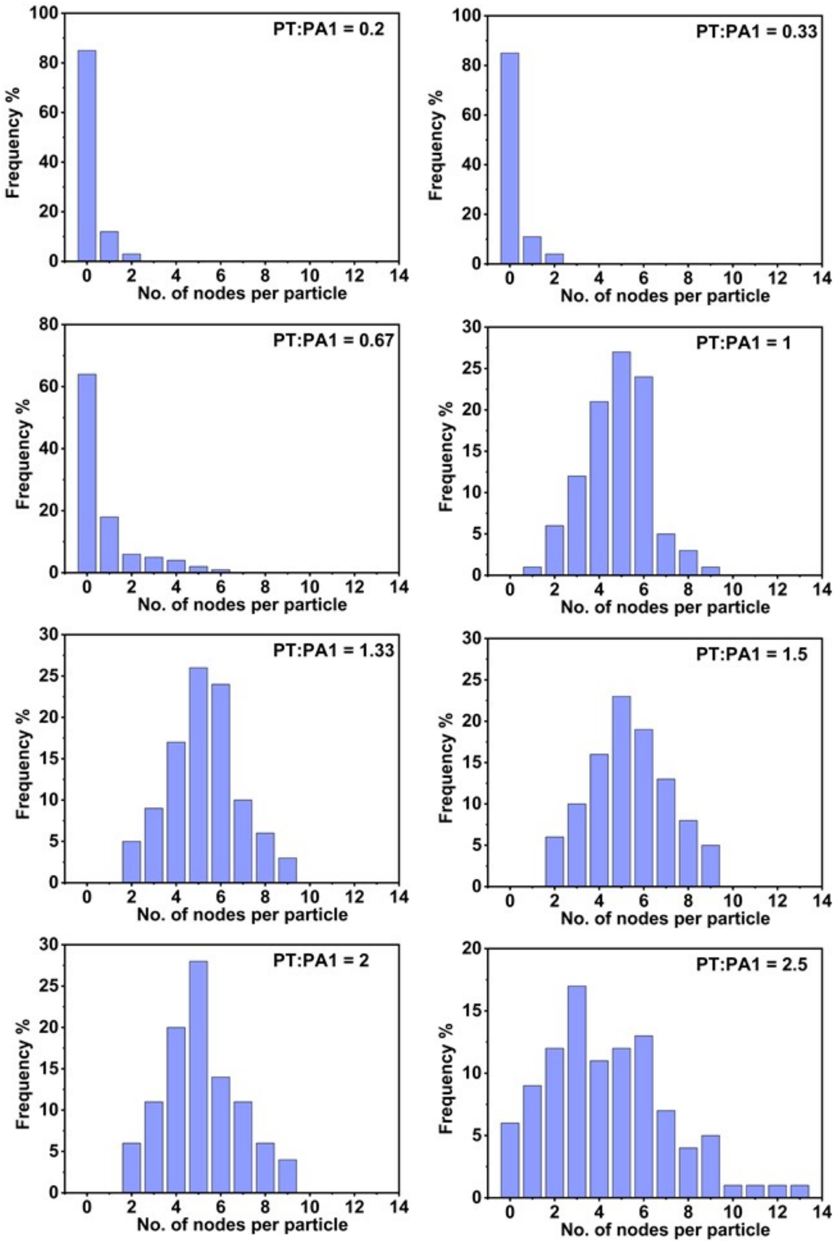

Figure S14: Histograms illustrating node distribtion on **PA1** after addition of **PT**. The equivalents of added **PT** is indicated in the top right hand side of each panel.

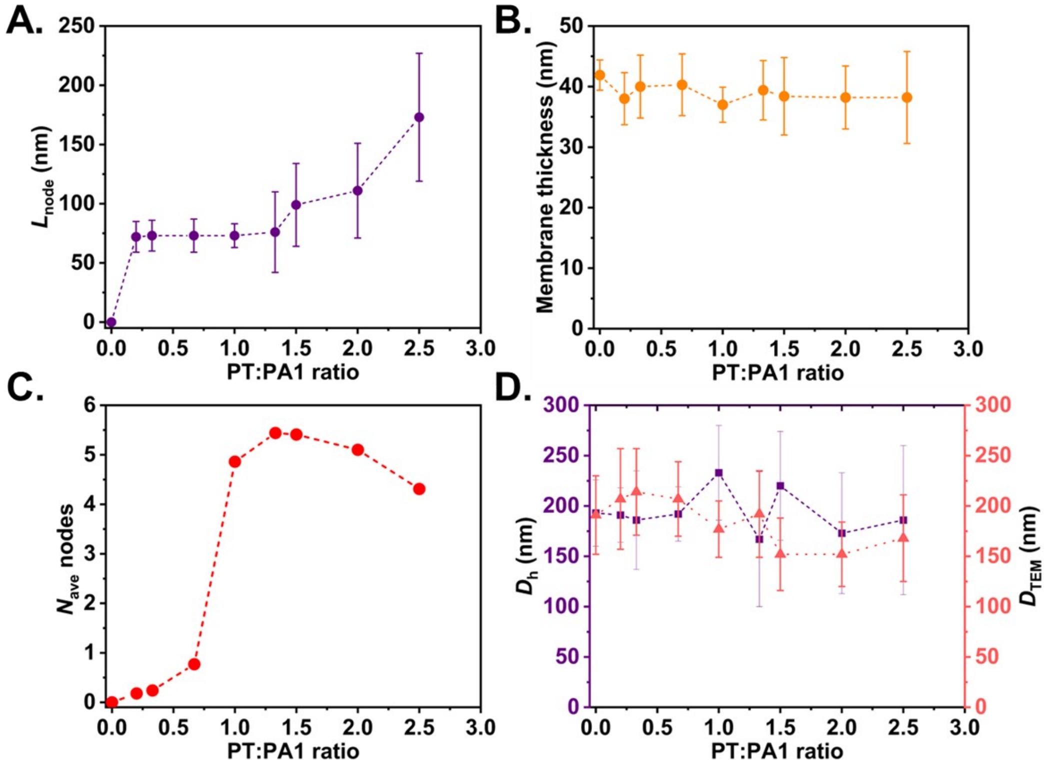

Figure S15: Quantitative analysis of **PA1** particles obtained after addition of different amounts of **PT**. (A) Length of the nodes. (B) Membrane thickness of **PA1**. (C) Number of nodes per particle. (D) Diameters obtained by DLS and TEM. The error bars represent standard deviation in each case.

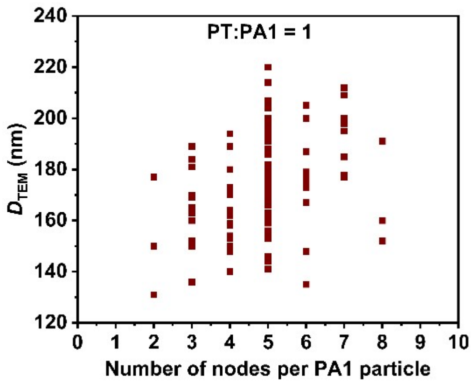

Figure S16: Correlation between number of nodes per particle and particle diameter (determined by TEM) for a sample of **PA1+PT** at 1:1 **PA2**:**PT** ratio.

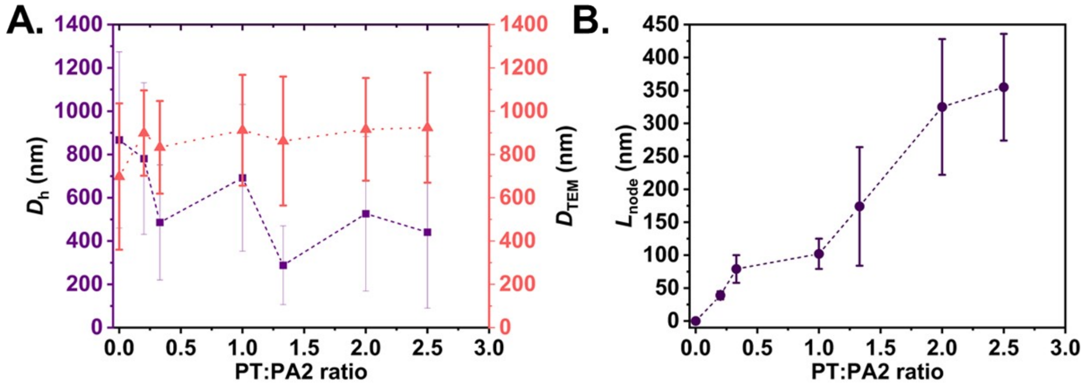

Figure S17: Quantitative analysis of **PA2** particles obtained after addition of different amounts of **PT**. (A) Diameters measured by DLS and TEM. (B) Length of the nodes at different ratios measured by TEM. The error bars represent standard deviation in each case.

Table S7: Summary of data for the addition of **PT** to **PA1** particles.

**Ratio of PT to PA1**	**0.2**	**0.33**	**0.67**	**1**	**1.33**	**1.5**	**2**	**2.5**
**D_h (nm)**	191 ± 27	186 ± 49	192 ± 27	233 ± 47	167 ± 67	220 ± 54	173 ± 60	186 ± 74
**D_{ave} (nm)**	207 ± 50	214 ± 43	207 ± 37	177 ± 28	192 ± 43	152 ± 36	152 ± 32	168 ± 43
**Node length (nm)**	72 ± 13	73 ± 13	73 ± 14	73 ± 10	76 ± 34	99 ± 35	111 ± 40	173 ± 54
**Nodes per particle**	0.18	0.24	0.77	4.86	5.44	5.41	5.10	4.31

Table S8: Summary of data from the addition of **PT** to **PA2** particles.

**Ratio of PT to PA2**	**0.2**	**0.33**	**1**	**1.33**	**2**	**2.5**
**D_h (nm)**	781 ± 350	486 ± 266	692 ± 339	288 ± 182	526 ± 357	408 ± 351
**D_{ave} (nm)**	899 ± 197	833 ± 214	912 ± 256	862 ± 298	916 ± 237	924 ± 254
**Node length (nm)**	39 ± 6	79 ± 21	102 ± 23	174 ± 90	325 ± 103	355 ± 81

Synthesis and Characterization of PNAM$_{40}$-*b*-PT^{Me}Am$_{20}$ (PT^{Me}$_{20}$) and PNAM$_{40}$-*b*-PAAm$_{20}$ (PA3)

Synthesis of 3-(3-methylthymin-1-yl)-propylacrylamide (T^{Me}Am)

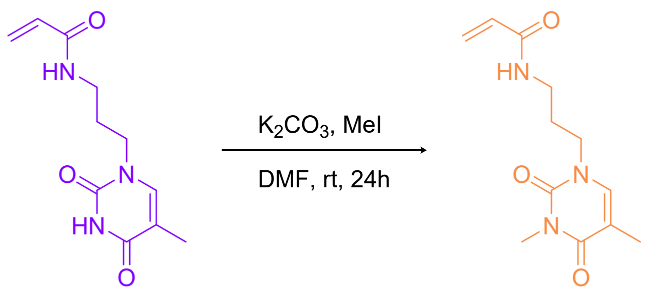

Scheme S5: Synthesis of 3-(3-methylthymin-1-yl)-propylacrylamide (**T^{Me}Am**).

T^{Me}Am was synthesized as reported previously.[1] A mixture of 3-(thymin-1-yl)propylacrylamide (TAm) (71 mg, 0.30 mmol, 1.0 eq), dry K_2CO_3 (66 mg, 0.48 mmol, 1.6 eq), and iodomethane (75 µL, 1.2 mmol, 4.0 eq) in anhydrous DMF (0.4 mL) was stirred at room temperature for 24 h and then diluted with ethyl acetate (20 mL), washed with water (2 × 20 mL), and dried with anhydrous Na_2SO_4. The solvent was removed under vacuum. The mixture was further purified by column chromatography with a mixture of CH_2Cl_2/CH_3OH (95:5) to give a white solid, T^{Me}Am (73 mg, 0.29 mmol, 97%). ^{1}H NMR (500 MHz, DMSO-*d$_6$*) δ: 8.13 (t, J = 5.0 Hz, CON***H***), 7.60 (s, 1H, pyrimidine-***H***), 6.18 (dd, J = 17.5, 10.5 Hz, 1H, CH$_2$-C***H***−CO), 6.08 (dd, J = 17.5, 2.0 Hz, 1H, C***H$_2$***-CH−CO), 5.60 (dd, J = 10.5, 2.0 Hz, 1H, C***H$_2$***-CH−CO), 3.70 (t, 2H, J = 7.5 Hz, C***H$_2$***-pyrimidine), 3.16 (s, 3H, OC−NC***H$_3$***), 3.14 (m, 2H, OC−HN−C***H$_2$***), 1.80 (s, 3H, C***H$_3$***-pyrimidine), 1.76 (m, 2H, OC−NH−CH$_2$−C***H$_2$***−CH$_2$-pyrimidine) ppm. ^{13}C NMR (125 MHz, DMSO-*d$_6$*) δ: 165.1, 163.8, 151.5, 140.4, 132.2, 125.5, 107.9, 47.1, 36.3, 29.0, 28.0, 13.1 ppm; HR-MS (m/z) found 274.1165, calc. 274.1162 [M + Na]$^+$.

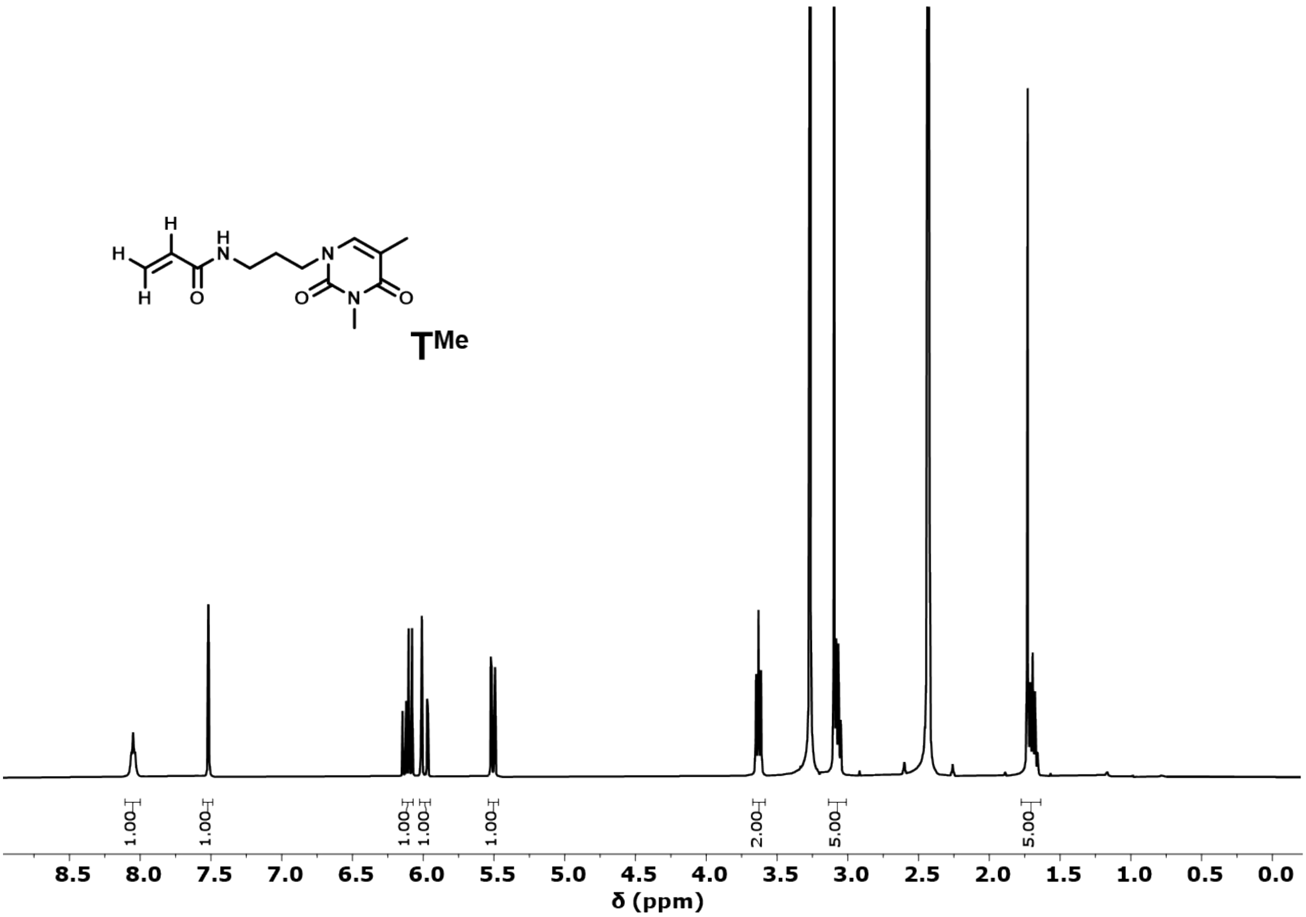

Figure S18: ^{1}H NMR spectrum of **T^{Me}Am** in DMSO-d_6 (400 MHz).

Synthesis of PNAM$_{40}$-*b*-PT^{Me}Am$_{20}$ diblock copolymer nano-objects by aqueous RAFT-mediated polymerization-induced self-assembly (PISA)

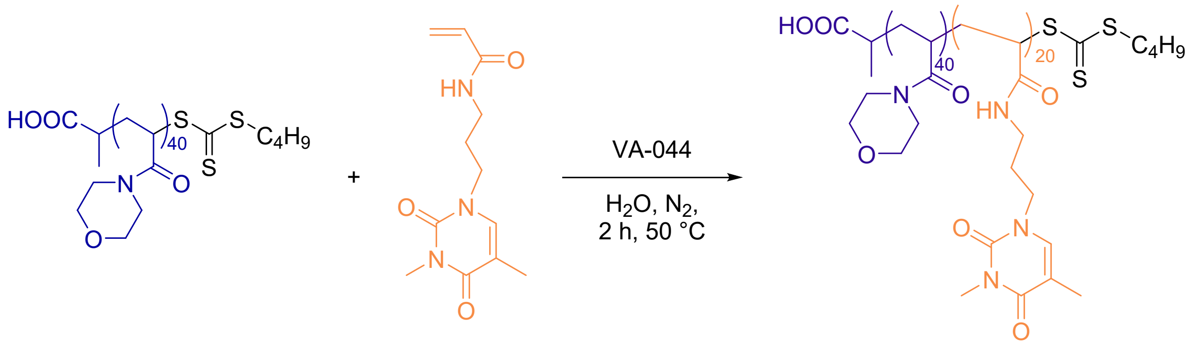

Scheme S6: Synthesis of PNAM$_{40}$-*b*-PT^{Me}Am$_{20}$ (**PT^{Me}**) diblock copolymer nano-objects.

A typical procedure to synthesise PNAM$_{40}$-*b*-PT^{Me}Am$_{20}$ diblock copolymer nano-objects at [solids] = 5% w/w *via* aqueous RAFT-mediated PISA is described. PNAM$_{40}$ macro-CTA (26.8 mg, 4.7×10^{-6} mol, 1 eq.), T^{Me}Am (25 mg, 9.5×10^{-5} mol, 20 eq.) and VA-044 (0.307 mg, 9.5×10^{-7} mol, 0.2 eq.) (158 µL of a stock solution containing 2 mg of VA-044 in 1 mL of water) were dispersed in deionized (DI) water (0.882 mL) and sealed in a 7 mL vial containing a magnetic stirrer bar. The resulting monomer-in-water solution was degassed by sparging with N_2(g) for 15 min. The sealed vial was heated at 50 °C with magnetic stirring for 2 h to ensure full monomer conversion. After this period, the reaction mixture was exposed to air and allowed to cool to room temperature.

Synthesis of PNAM$_{40}$-*b*-PAAm$_{20}$ diblock copolymer nano-objects by aqueous RAFT-mediated polymerization-induced self-assembly (PISA)

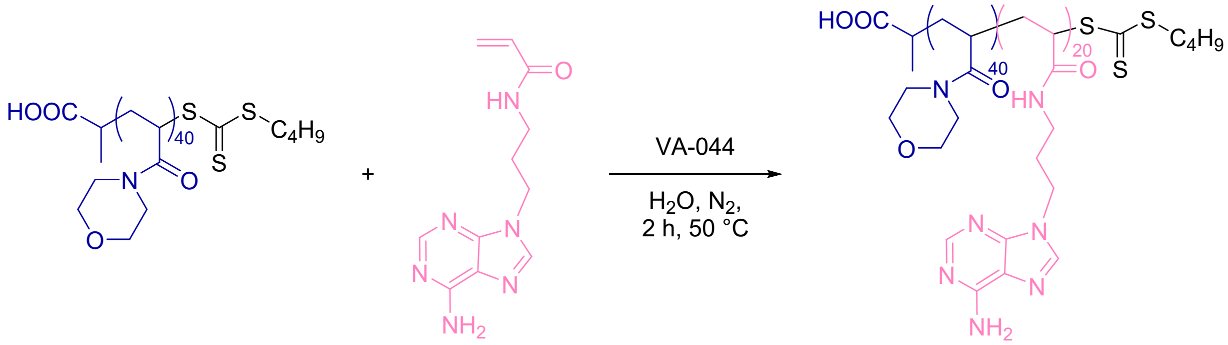

Scheme S7: Synthesis of PNAM$_{40}$-*b*-PAAm$_{20}$ (**PA3**) diblock copolymer nano-objects.

A typical procedure to synthesise PNAM$_{40}$-*b*-PAAm$_{20}$ diblock copolymer nano-objects at [solids] = 5% w/w *via* aqueous RAFT-mediated PISA is described. PNAM$_{40}$ macro-CTA (28 mg, 4.96 × 10^{-6} mol, 1 eq.), AAm (25 mg, 9.92 × 10^{-5} mol, 20 eq.) and VA-044 (0.321 mg, 9.92 × 10^{-7} mol, 0.2 eq.) (158 µL of a stock solution containing 2 mg of VA-044 in 1 mL) were dispersed in deionized (DI) water (1.07 mL) and sealed in a 7 mL vial containing a magnetic stirrer bar. The resulting monomer-in-water solution was degassed by sparging with N_2(g) for 15 min. The sealed vial was heated at 50 °C with magnetic stirring for 2 hours to ensure full monomer conversion. After this period, the reaction mixture was exposed to air and allowed to cool to room temperature.

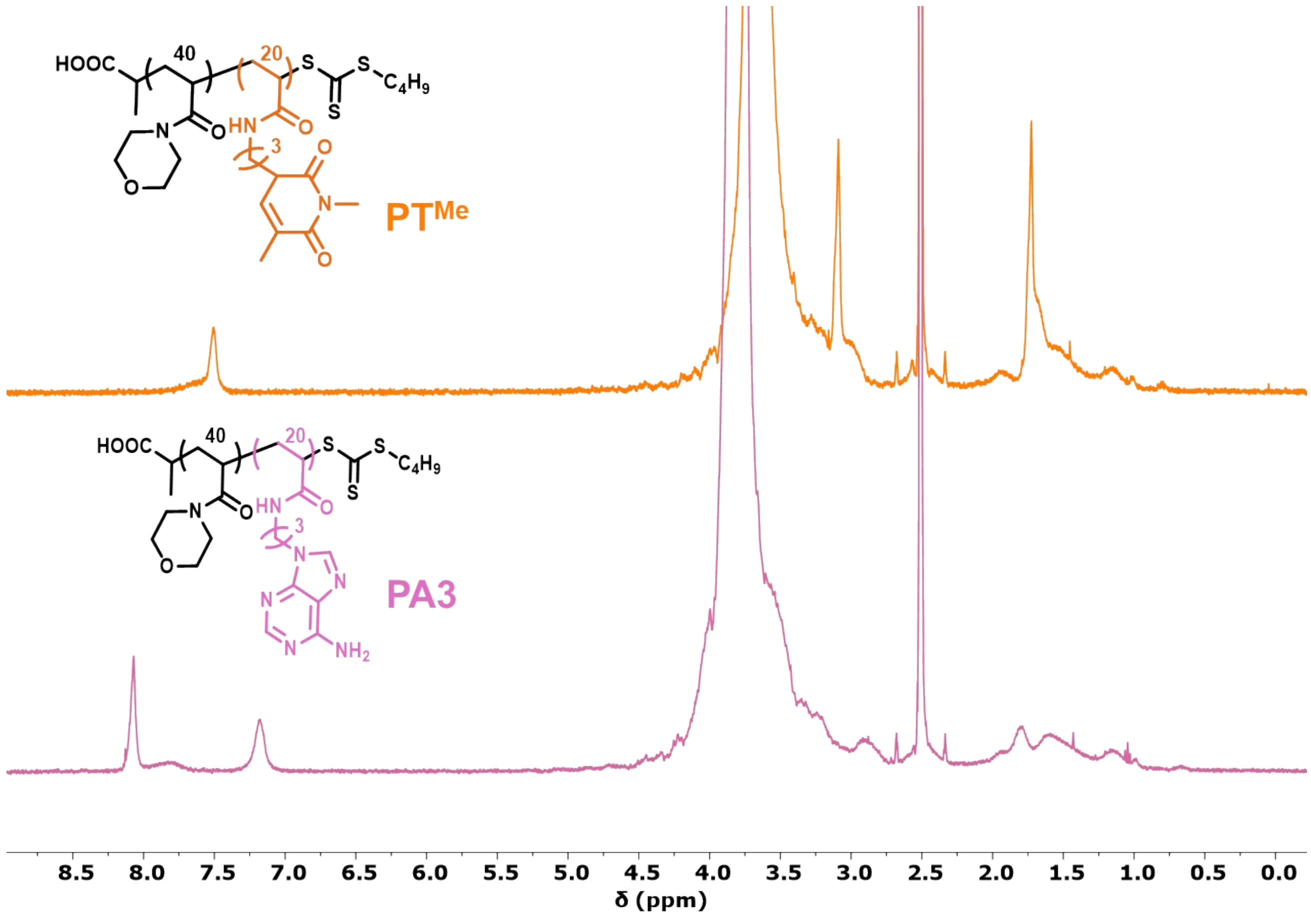

Figure S19: ^{1}H-NMR spectra of PNAM$_{40}$-*b*-PT^{Me}Am$_{20}$ (**PT^{Me}**) and PNAM$_{40}$-*b*-PAAm$_{20}$ (**PA3**) obtained by aqueous RAFT-mediated PISA recorded in DMSO-d_6 (400 MHz).

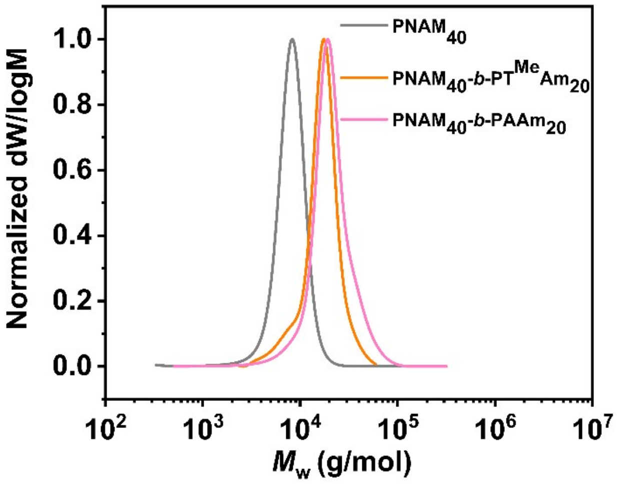

Figure S20: Normalized molecular weight distributions of $PNAM_{40}$ macro-CTA, $\mathbf{PT^{Me}}$ ($PNAM_{40}$-*b*-$PT^{Me}Am_{20}$) and **PA3** ($PNAM_{40}$-*b*-$PAAm_{20}$) (DMSO + 0.1% w/w LiBr as eluent, PMMA standards).

Table S9: Characterization data for $\mathbf{PT^{Me}_{20}}$ ($PNAM_{40}$-*b*-$PT^{Me}Am_{20}$) and **PA3** ($PNAM_{40}$-*b*-$PAAm_{20}$).

Polymers	% Conv.[a]	$M_{n, NMR}$[b] / kDa	$M_{n, SEC}$[c] / kDa	$Đ_{M, SEC}$[c]
$PNAM_{40}$-*b*-$P^{Me}TAm_{20}$	> 99	11.1	14.2	1.25
$PNAM_{40}$-*b*-$PAAm_{20}$	> 99	10.7	18.2	1.24

[a]Monomer conversion calculated from ^{1}H-NMR spectroscopy in D_2O. [b]Calculated from conversion. [c]M_n and $Đ_M$ values calculated from PMMA standards using DMSO + 0.1% w/w LiBr as the eluent.

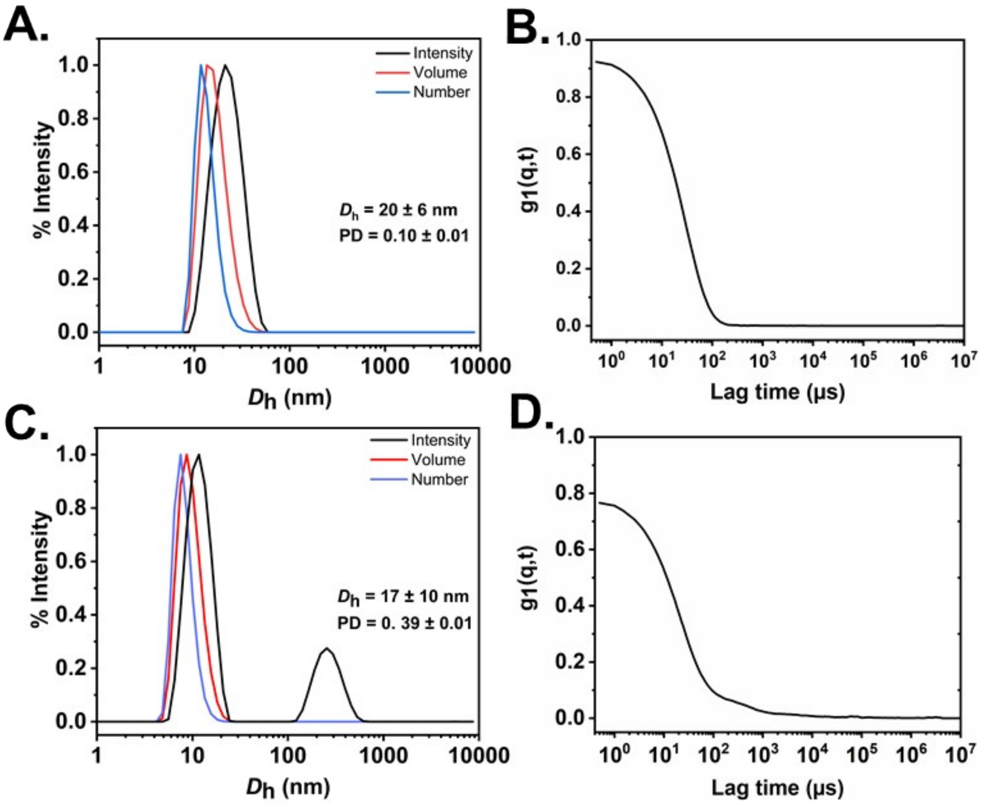

Figure S21: DLS particle size distribution and corresponding correlogram graph results of **PA3** (A and B) and **PT^{Me}** (C and D).

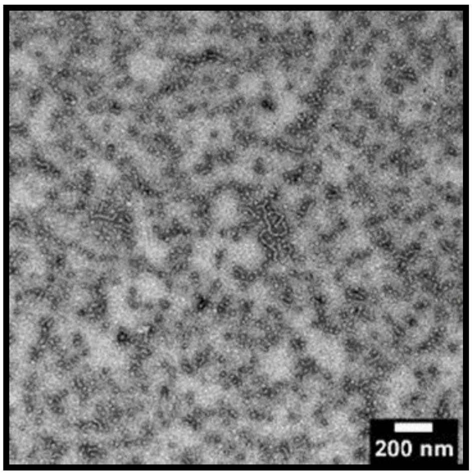

Figure S22: TEM image of **PA3** ($PNAM_{40}$-*b*-$PAAm_{20}$) diblock copolymer nanoparticles. The sample was stained with 1 wt % uranyl acetate (UA) solution.

Supplementary Characterization Data for Morphological Transformation Control Experiments: Addition of non-complementary block copolymers to a PA1 solution.

PT^{Me} and **PA3** were mixed with separate solutions of **PA1** at a ratio of 1:1, and the resulting nanoparticles were analysed by DLS and TEM. Initial seed particle solutions of **PA1** were prepared at a concentration of 0.5 mg mL^{-1} in water, whilst **PA3** and **PT^{Me}** were diluted with water to 5 mg mL^{-1}. **PA3** or **PT^{Me}** were added to **PA1** at a ratio of 1:1 and stirred for 2 h.

Table S10: Volumes of solutions **PA3** and **PT^{Me}** required for mixing with **PA1** at a 1:1 ratio.

**Experiment**	**V_{PA1} (µL)**	**V_{PA3} /$V_{PT^{Me}}$ (µL)**
**PA1** + **PA3**	250	4.75
**PA1** + **PT^{Me}**	250	4.85

Table S11: Summary of DLS and TEM characterization data of control experiments.

Particles	D_h (nm)[a]	PD[a]	D_{ave} (nm)[b]	Morphology[c]	Comments
**PA1 + PA3**	179 ± 25	0.02 ± 0.02	178 ± 20	P	No change
**PA1 + PT^{Me}**	194 ± 27	0.02 ± 0.01	184 ± 23	P	No change

[a]D_h and PD values measured by DLS (the error shows standard deviation from three repeat measurements). [b]D_{ave} values of polymersomes obtained from TEM analysis. [c]Morphologies observed from dry-state TEM imaging, using 1 wt % uranyl acetate (UA) solution for staining (Key: P – polymersomes).

Supplementary Characterization Data for confocal microscopy experiments

Synthesis of fluorescent $PNAM_{40}$

$PNAM_{40}$ macroCTA was tagged with a green (**BODIPY FL**) or red (**BODIPY 630/650**) dye as shown in Supplementary Scheme S8 and S9 by modifying the carboxylic acid end group of $PNAM_{40}$ macroCTA with amine-containing dye molecules. A typical procedure is as follows: 100 mg of $PNAM_{40}$ macroCTA was dissolved in 3 mL of water. 2.5 mg 1-Ethyl-3-(3-dimethylaminopropyl)carbodiimide (EDC) was added in the solution and stirred for at least 5 minutes. Stock solutions of BODIPY FL or BODIPY 630/650 were prepared in advance at a dilution of 5 mg mL^{-1}. 140 µL was added for BODIY FL and 200 µL for BODIPY 630/650 solutions respectively. The resulting solution was then stirred at room temperature for 48h. The solutions were then dialyzed against water for at least 3 days with 2 water changes per day. The resulting polymers, **mCTA^{Gr}** and **mCTARd**, were freeze dried and further characterized by ^{1}H NMR and SEC analysis.

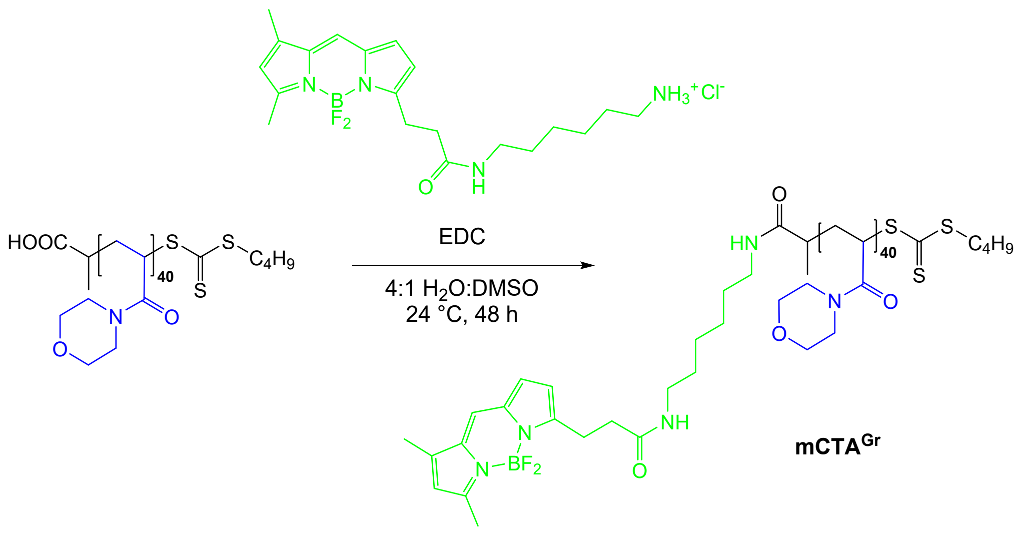

Scheme S8: Synthesis of poly(4-acryolylmorpholine)$_{40}$ ($PNAM_{40}$) **mCTA^{Gr}** with BODIPY FL.

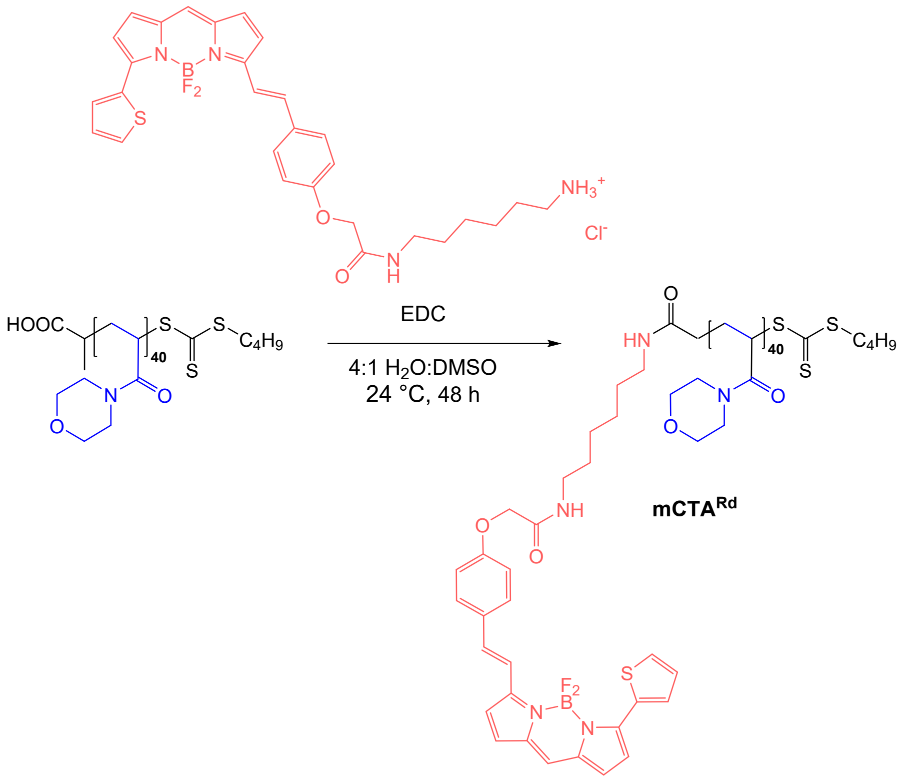

Scheme S9: Synthesis of poly(4-acryolylmorpholine)$_{40}$ (PNAM$_{40}$) **mCTARd** with BODIPY 630/650.

Synthesis of fluorescent PNAM$_{40}$-*b*-PTAm$_{20}$Rd (PTRd) and PNAM$_{40}$-*b*-PAAm$_{222}$^{Gr} (PA2^{Gr}) nano-objects

RAFT-PISA with fluorescent **mCTA^{Gr}** and **mCTARd** was performed analogously to the untagged nano-objects **PT** ([solids] = 5% w/w) and **PA2** ([solids] = 15% w/w), as per schemes S10 and S11.

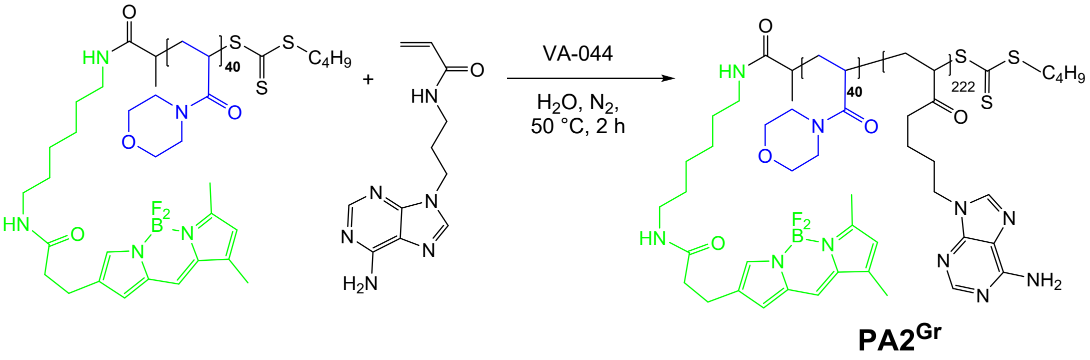

Scheme S10: Synthesis of **PA2^{Gr}** diblock copolymer nano-objects by aqueous RAFT-mediated polymerization-induced self-assembly (PISA).

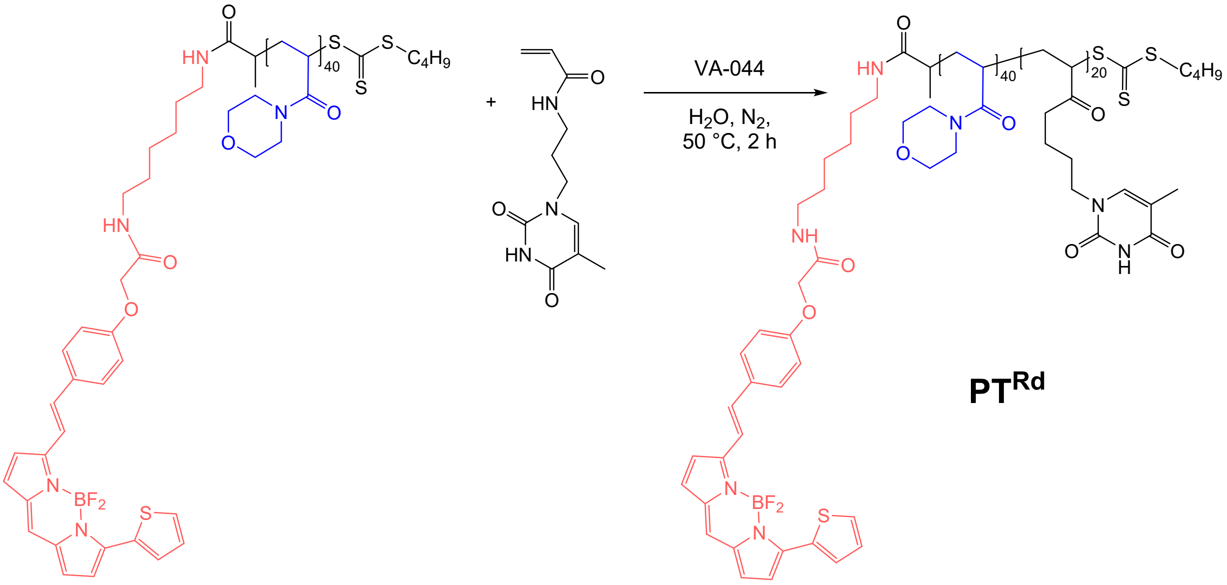

Scheme S11: Synthesis of **PTRd** diblock copolymer nano-objects by aqueous RAFT-mediated polymerization-induced self-assembly (PISA).

Supplementary Characterization Data for fluorescent PNAM40, PA2Gr and PT^{Rd}

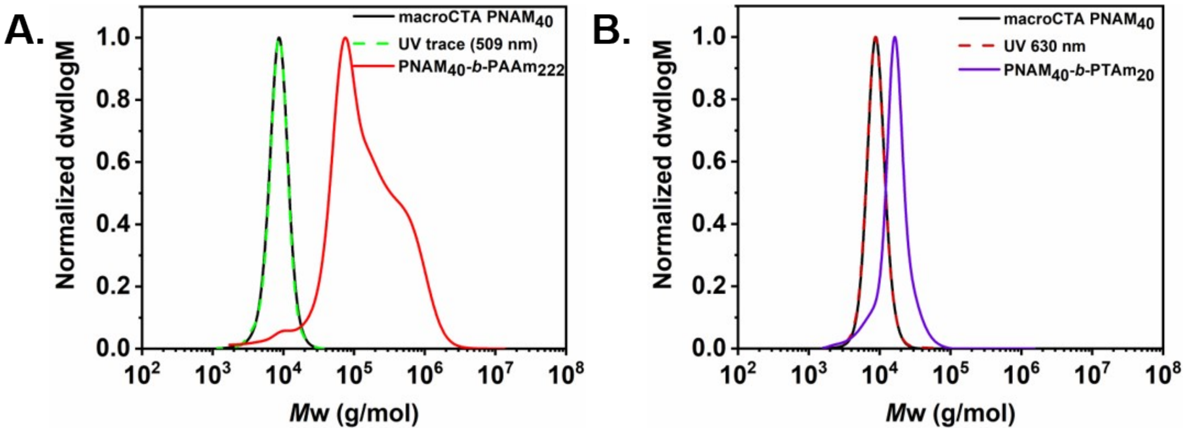

Figure S23: (A) Combined SEC traces of $PNAM_{40}$ **$mCTA^{Gr}$** and **$PA2^{Gr}$** and (B) combined SEC traces of $PNAM_{40}$ **$mCTA^{Rd}$** and **PT^{Rd}** (DMSO + 0.1% w/w LiBr as eluent, PMMA standards).

Table S12: Molecular characteristics of $PNAM_{40}$-*b*-$PTAm_{20}$ (**PT**) prepared via aqueous RAFT-mediated PISA, as determined by ^{1}H-NMR spectroscopy and SEC analysis.

Polymer	Targeted DP	% Conv.[a]	$M_{n,NMR}$[b] /kDa	$M_{n,SEC}$[c] /kDa	$Đ_{M,SEC}$[c]
**$PNAM_{40}$ $mCTA^{Gr}$**	40	> 99	5.6	7.9	1.13
**PNAM40 $mCTA^{Rd}$**	40	> 99	5.6	8.4	1.10
**PT_{20}^{Rd}**	20	> 99	10.3	13.8	1.30
**$PA2^{Gr}$**	300	70	60.1	62.1	3.68

[a]Monomer conversion calculated from ^{1}H-NMR spectroscopy in DMSO-d_6 (400 MHz). [b]Calculated from conversion. [c]M_n and $Đ_M$ values calculated from DMSO + 0.1% w/w LiBr as the eluent with PMMA standards.

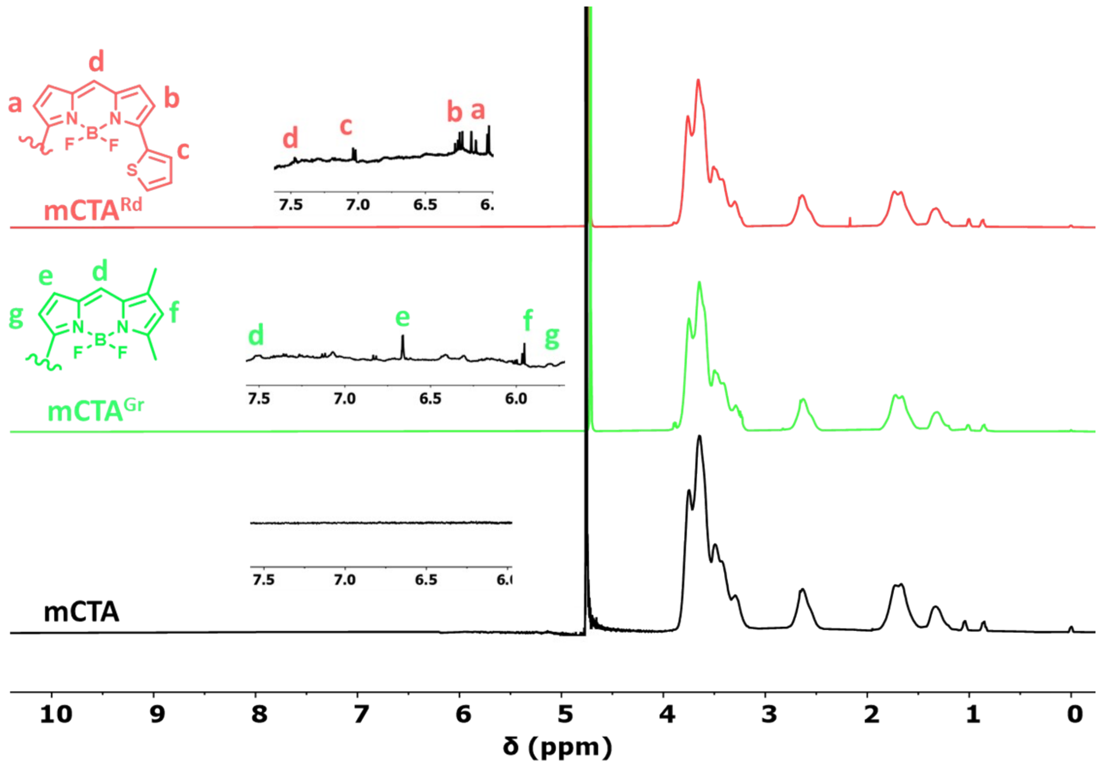

Figure S24: ^{1}H NMR spectra of $PNAM_{40}$ **mCTA**$^{\mathbf{Rd}}$, **PNAM$_{40}$ mCTA**$^{\mathbf{Gr}}$, and unmodified **PNAM$_{40}$** recorded in DMSO-d_6 (500 MHz).

Table S13: Summary of DLS and TEM characterization data of **PT**$^{\mathbf{Rd}}$ and **PA2**$^{\mathbf{Gr}}$ block copolymer nano-objects obtained by aqueous RAFT-mediated PISA.

Particle	D_h (nm)^a	PD^a	D_{ave} (nm)^b	Morphology^C
**PT**$^{\mathbf{Rd}}$	24 ± 19	0.6 ± 0.04	-	S+W
**PA2**$^{\mathbf{Gr}}$	781 ± 366	0.22 ± 0.01	616 ± 216	P

$^a D_h$ and PD values measured from DLS analysis (the error shows the standard deviation from 3 repeat measurements). $^b D_{ave}$ values obtained from TEM analysis. ^{c}Morphologies observed from dry-state TEM imaging, using 1% w/w uranyl acetate (UA) solution for staining (Key: S – spheres, W – worms and P – polymersomes).

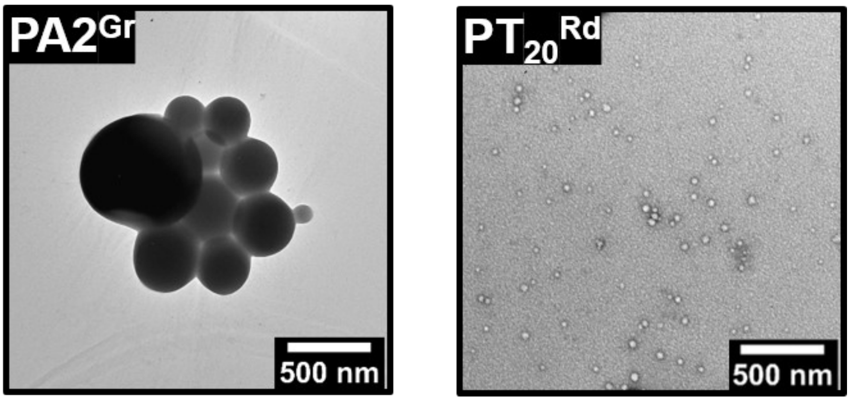

Figure S25: Dry-state TEM images for **PA2^{Gr}** and **PTRd.** Dry-state samples were stained using 1 wt % uranyl acetate (UA) solution.

Confocal microscopy of PA2+PT particles

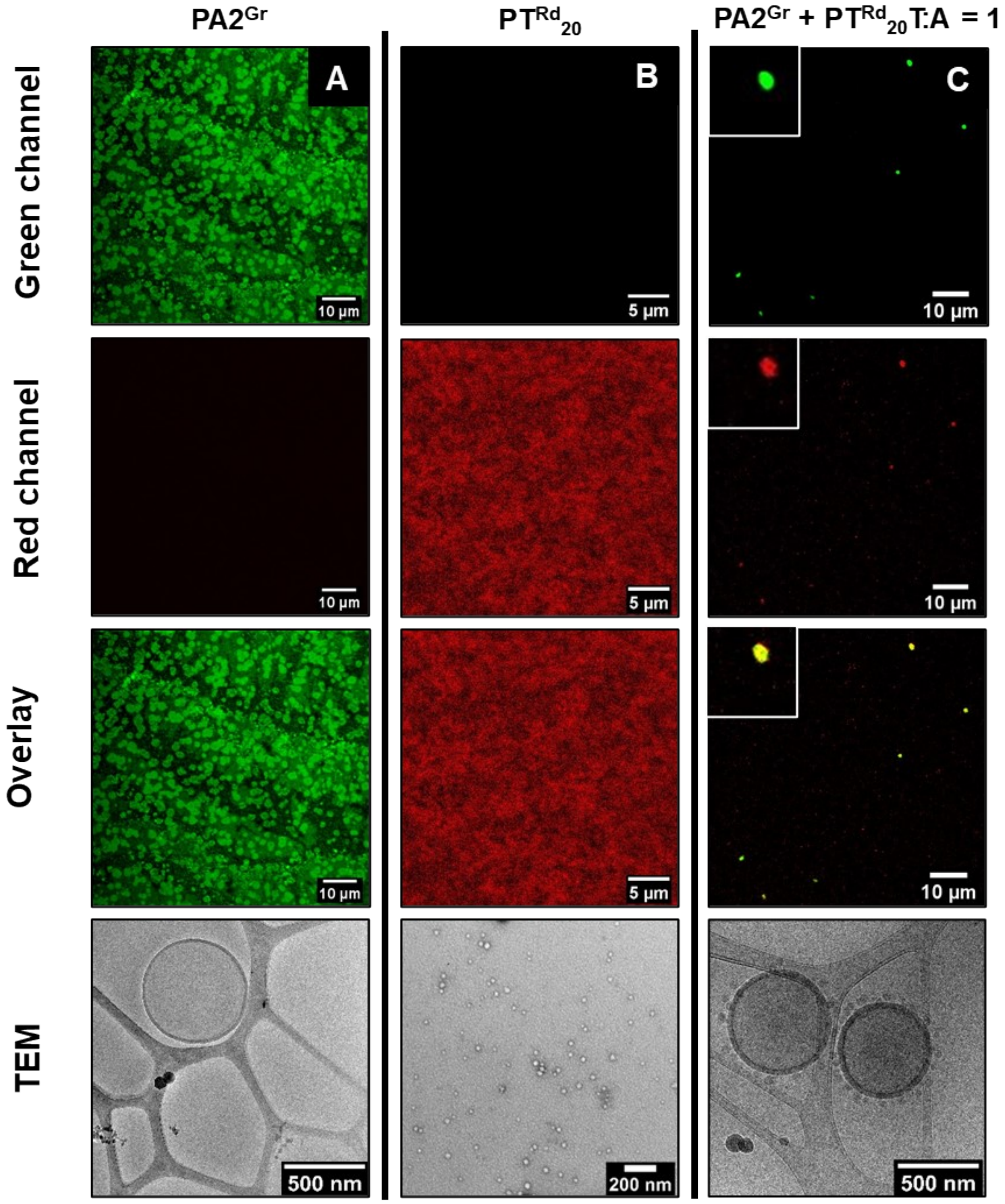

Figure S26: Confocal microscopy images for pure $\mathbf{PA2^{Gr}}$ (A), $\mathbf{PT^{Rd}}$ (B) and $\mathbf{PA2^{Gr}}$+ 1 eq. $\mathbf{PT^{Rd}}$ (C). The scale bars are indicated in the images and the size of the insets is 10 µm × 10 µm. Representative cryo-TEM ($\mathbf{PA2^{Gr}}$ and $\mathbf{PA2^{Gr}}$ + $\mathbf{PT^{Rd}}$) and dry state TEM ($\mathbf{PT^{Rd}}$) images are shown on the bottom row.

References

1. Z. Hua, J. R. Jones, M. Thomas, M. C. Arno, A. Souslov, T. R. Wilks and R. K. O'Reilly, *Nat. Commun.*, 2019, **10**, 5406.
